# Unsupervised learning of aging principles from longitudinal data

**DOI:** 10.1038/s41467-022-34051-9

**Published:** 2022-11-01

**Authors:** Konstantin Avchaciov, Marina P. Antoch, Ekaterina L. Andrianova, Andrei E. Tarkhov, Leonid I. Menshikov, Olga Burmistrova, Andrei V. Gudkov, Peter O. Fedichev

**Affiliations:** 1 Gero PTE. LTD., 409051 Singapore, Singapore; 2 Department of Pharmacology and Therapeutics, Roswell Park Comprehensive Cancer Center, Buffalo, NY USA; 3 Genome Protection, Inc., Buffalo, NY USA; 4 Department of Cell Stress Biology, Roswell Park Comprehensive Cancer Center, Buffalo, NY USA

**Keywords:** Biomarkers, Ageing, Computational models, Machine learning

## Abstract

Age is the leading risk factor for prevalent diseases and death. However, the relation between age-related physiological changes and lifespan is poorly understood. We combined analytical and machine learning tools to describe the aging process in large sets of longitudinal measurements. Assuming that aging results from a dynamic instability of the organism state, we designed a deep artificial neural network, including auto-encoder and auto-regression (AR) components. The AR model tied the dynamics of physiological state with the stochastic evolution of a single variable, the “dynamic frailty indicator” (dFI). In a subset of blood tests from the Mouse Phenome Database, dFI increased exponentially and predicted the remaining lifespan. The observation of the limiting dFI was consistent with the late-life mortality deceleration. dFI changed along with hallmarks of aging, including frailty index, molecular markers of inflammation, senescent cell accumulation, and responded to life-shortening (high-fat diet) and life-extending (rapamycin) treatments.

## Abstract (web summary)

Biomarkers of age and frailty may aid in understanding the aging process, predicting lifespan or health span and in assessing the effects of anti-aging interventions. Here, the authors show that combining physics-based models and deep learning may enhance understanding of aging from big biomedical data, observe effects of anti-aging interventions in laboratory animals, and discover signatures of longevity.

## Introduction

Aging manifests itself on multiple levels of the organism organization. Accordingly, diverse sets of physiological state variables, such as DNA methylation patterns or blood composition markers^1–5^, can be used to quantify aging. State-of-the-art biomarkers of aging came out from supervised models that require chronological age or mortality age as labels for training (see, e.g., the most recent^6^). Alternatively, the number of health deficits accumulated^7^ or composite frailty indices^8,9^ correlate with the disease burden and remaining lifespan of humans and animals^9,10^ and find applications in laboratory experiments and clinical trials of anti-aging interventions^3,11,12^. Future use of aging biomarkers will depend on a better understanding of the connection between aging hallmarks, biological age predictors, frailty indices, and the remaining lifespan.

Here, we demonstrate a combination of modern machine learning techniques and approaches borrowed from the dynamic systems theory working together to identify quantitative rules governing manifestations of aging and their relation to all-cause mortality in big longitudinal biomedical data. We start by presuming that aging is a particular case of the dynamics of a complex system unfolding near a bifurcation or a tipping point on the boundary of a dynamic stability region. Under the circumstances, the organism state fluctuations should be driven by the dynamics of a very few, if not a single collective feature having the meaning of the order parameter corresponding to the unstable phase^13–15^. Examples of such dimensionality reduction in biological sciences include development^16^, aging, and mortality acceleration^5,17,18^.

We built upon this theoretical concept and produced a deep artificial neural network composed of a denoising autoencoder (AE) and an auto-regressive (AR) model — a computational metaphor for the stochastic dynamics of the order parameter. To test the approach, we used the network to produce a descriptor of aging based on complete blood count (CBC) measurements from the largest open-access source of phenotypic data, the Mouse Phenome Database (MPD)^19^. The network output variable, hereinafter referred to as the dynamic frailty indicator (dFI), is the best numerical approximation for the order parameter associated with the organism state disintegration and hence aging from any given data. We demonstrated that the dynamics of dFI drives the mortality acceleration and explain late-life deviations from Gompertz mortality law. The dFI exhibited the most desirable properties of a biological age marker: it increased exponentially with age, predicted the remaining lifespan of the animals, correlated with multiple hallmarks of aging, and responded to interventions known to accelerate (high-fat diet) and decelerate (rapamycin) aging in mice.

## Results

### Principal component analysis of CBC in the MPD

We used the largest publicly available longitudinal phenotypic data source, the MPD^19^. To achieve the best possible compatibility with earlier studies, we scanned the database records to maximize the number of available measurements common to those used in constructing the physiological frailty index (PFI) in ref. 3. As a result, we chose a subset of 12 CBC measurements from nine datasets, altogether including 6693 animals (see Supplementary Data 1 for the full list of datasets used for training the models in this work).

**Fig. 1:**
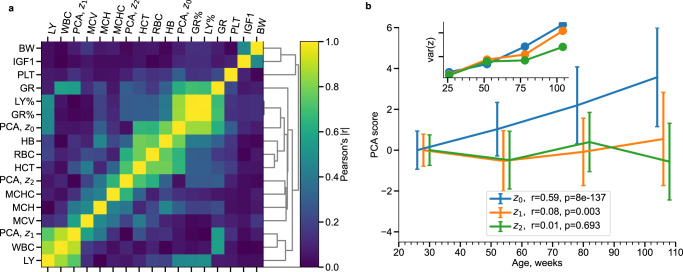
Hierarchical clustering and Principal Component (PC) Analysis of complete blood count (CBC) measurements from The Mouse Phenome Database. **a** Clustering of CBC features and PC scores in the training dataset. The colors represent Pearson’s correlation coefficient (absolute value) as indicated by the scale on the right. **b** The graphs represent the average of the PC scores in subsequent age groups (the error bars are standard deviations). The inset shows that the variance for all PC scores increases with age. Two-sided *p* values were calculated for the Pearson correlation coefficient in the sample size of *n* = 1448 animals.

To visualize the 12-dimensional CBC data from the MPD, we performed principal component analysis (PCA). PCA of the MPD slice representing fully grown animals (exceeding the age of 25-week-old, see Methods for the details regarding the relevant age-range determination) turned out to be particularly simple. In this case, most of the variance in the data (31%) is explained by the first PC score, *z*_0_, with the subsequent PC scores (*z*_1_, *z*_2_, etc., each explaining 19%, 16%, etc. of the total variance in the data, respectively). The hierarchical clustering of the CBC features suggested that the first two PC scores could be predominantly connected with the red and white blood cell counts, as shown in Fig. 1a, respectively.

Only the first PC score, *z*_0_, demonstrated an appreciable correlation with the chronological age (the corresponding Pearson’s correlation coefficients were *r* = 0.59 (*p* < 10^−5^) and *r* = 0.08 (*p* = 0.003) for the first two PC scores, respectively (Fig. 1b). The variance of the PC scores increased with age (see the inset in Fig. 1b), which is a signature of a stochastic process.

### Aging and critical dynamics of the organism’s state (the summary of theoretical results)

The association of the fluctuations along the dominant PC with the slowest dynamic process (aging, in our case) has deep roots in the dynamic systems theory and critical phenomena^14^. The transition from stability to instability in networks with the network graphs not possessing specific symmetries is typically associated with co-dimension 1 (or saddle-node) bifurcations^20,21^. Such transitions are characterized by the loss of stability along a single direction in the state vector space, approximately coinciding with the first principal component. In contrast, contributions from all other principal components remain stable (and hence age-independent). Accordingly, over sufficiently long timescales, the fluctuations of physiological indices (such as CBC features), *x*_*i*_, are expected to follow the dynamics of the order parameter, *z*, associated with the instability: *x*_*i*_ ≈ *b*_*i*_*z* + *ξ*_*i*_. Here *ξ*_*i*_ is noise, *b*_*i*_ is a vector, and the integer index *i* enumerates the measured features.

Close to the tipping point, the dynamics of the physiological state is slow and approximately linear. Hence the variable *z* satisfies the stochastic Langevin equation with the higher-order time derivative terms neglected:

1 $$\dot{z}=\alpha z+g{z}^{2}+f.$$

Here the linear term, *αz*, on the right side of the equation represents the effect of the regulatory network stiffness governing the organism’s responses to small stresses and producing slight deviations of the organism state from its most stable position. The following term, *gz*^2^, represents the lowest-order non-linear coupling effects of regulatory interactions.

The stochastic forces *f* represent external stresses and the effects of endogenous factors not described by the effective Eq. (1). Naturally, we assume that random perturbations of the organism state are serially uncorrelated, so that $\langle f(t)f({t}^{\prime})\rangle \sim B\delta ({t}^{\prime}-t)$, where *B* is the power of the noise, *δ* is the Dirac’s delta-function, and 〈…〉 stands for averaging along the aging trajectory. Eq. (1) is a mathematical relationship between the rate of change of the organism state variable, $\dot{z}=dz/dt$, on the left side of the equation, and the effects of deterministic (*αz*, *gz*^2^) and stochastic forces (*f*), on the right side.

Depending on the sign of the stiffness coefficient, *α*, the organism state may be dynamically stable (if *α* < 0) or unstable (if *α* > 0). In the latter case, minor deviations of the organism state get amplified exponentially over time, so no equilibrium is possible. In this case, the solution of Eq. (1) describes an aging organism. Typically, *α* is small, and hence, the evolution of the physiological indices exhibits hallmarks of critically: the dynamics of the order parameter are slow (critical slowing down), whereas the fluctuations of the physiological state following the variations in *z* are large, ${z}^{2} \sim B\exp (2\alpha t)/2\alpha \gg 1$ (critical fluctuations).

Very early in life, the deviations from the critical point are small, and the evolution of the organism state is dominated by diffusion. Later in life, the linear term takes over such that the deviations from the youthful state accelerate exponentially:

2 $$z\, \approx \, \bar{z}\exp (\alpha t)+{z}_{0},$$

where $\bar{z} \sim {(B/\alpha )}^{1/2}$ and *z*_0_ are constants representing the accumulated early effects of random and deterministic forces, respectively.

Finally, once the order parameter is sufficiently large, *z* ≳ *Z* = *α*/*g*, the non-linear terms take over, the disintegration of the organism state proceeds at a rate greater than exponential, and the animal dies in a finite time. Eq. (1) can be solved to obtain the simple analytical expression for the fraction of animals surviving up until the age *t*: $S(t)\, \approx \,{{{{{{{\rm{Erf}}}}}}}}(Z/\bar{z}\exp (-\alpha t))$, where Erf(x) is the error function^18^. The theoretical survival function is different from that of the Gompertz mortality law implying the exponential acceleration of mortality at all ages.

The all-cause mortality in the model, $M(t)=-\dot{S}(t)/S(t)$, increases quickly at first in the diffusion-dominated regime and then slows down at the age, approximately coinciding with the average lifespan $\bar{t}=1/\alpha \log (Z/\bar{z})$. In the eldest animals, the disagreement with the Gompertz mortality law is the strongest: the mortality in the model decelerates and reaches the plateau at $M(t\, \gg \,\bar{t})\, \approx \,\alpha$.

Finally, we observe, that the sample co-variance matrix of the organism state variables in the unstable regime is dominated by the dynamics of the order parameter:

3 $$\langle {x}_{i}(t){x}_{j}(t)\rangle\, \approx \,{b}_{i}{b}_{j}\frac{B}{2\alpha }\exp (2\alpha t),$$

where 〈…〉 stands for sample average. In the weak nonlinearity limit, $\alpha \bar{t}\, \gg \,1$, PCA of such signal would reveal the loading vector corresponding to the first PC score coinciding with *b*_*i*_. The first PC score *z*_0_ = (*b*, *x*) is then the approximation to the order parameter *z* ≈ *z*_0_ itself.

### Deep learning aging descriptors from longitudinal data

Aging at criticality may be a useful theoretical concept. However, it does not provide a practical algorithm for identifying the order parameter other than an approximate application of PCA. To characterize the aging process from the measurements, we analyzed the longitudinal aging trajectories of CBC measurements from 6,693 samples in nine MPD datasets (see Methods and Supplementary Data 1) with the help of an artificial neural network. The algorithm included a combination of a deep auto-encoder (AE) for the dimensionality reduction and a simple AR model for the fit of the network representations of the subsequent measurements onto solutions of Eq. (1) (AE–AR; see Methods).

The AR problem’s solution, *z*, is the output of the algorithm and is the best fit estimation of the order parameter associated with the disintegration of the organism state and hence aging from the available data. Below we establish the associations of *z* with hallmarks of aging, frailty, and mortality. Accordingly, we chose to refer to *z* as the dFI.

**Fig. 2:**
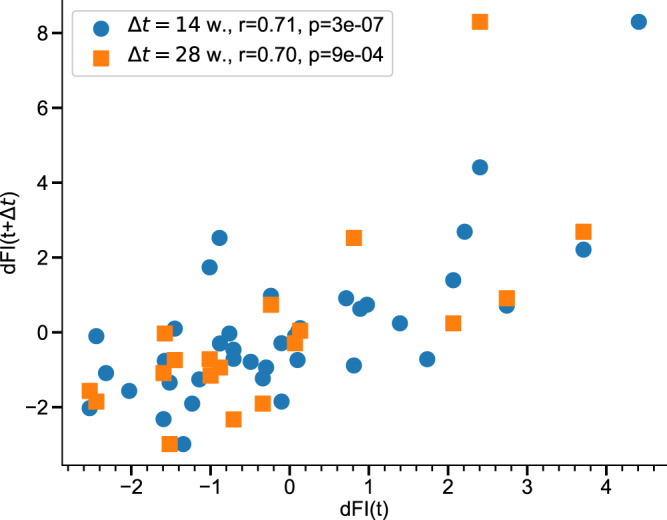
Auto-correlation property of the dynamic frailty indicator (dFI). Correlation between age-adjusted dFI values across sampling intervals Δ*t* of 14 (blue circles, animals *n* = 40) and 28 (orange squares, animals *n* = 19) weeks in the validation MA0072 dataset. Two-sided *p* values were calculated for the Pearson correlation coefficient.

The performance of the AR model was demonstrated by plotting the auto-correlation property of dFI, which is the correlation between dFI values measured along aging trajectories of the same mice at age points separated by 14 and 28 weeks in the test dataset MA0072 (Fig. 2). The corresponding Pearson correlation coefficients between the respective age-adjusted dFI estimations were *r* = 0.71 (*p* < 0.001) and *r* = 0.70 (*p* < 0.001). The dFI auto-correlations were better than the auto-correlations of the first PC score *z*_0_ for the same mice, see Supplementary Fig. 2; the corresponding Pearson’s correlation values were *r* = 0.55 (*p* < 0.001) and *r* = 0.71 (*p* < 0.001) for 14- and 28-week time lags, correspondingly.

A semi-quantitative hierarchical clustering of the CBC features’ co-variances in the test dataset produced correlations across the features associated with the immune system (white blood cell counts and the related quantities), metabolic rate/oxygen consumption (red blood cell counts and hemoglobin concentrations), and an apparently independent subsystem formed by platelets (Supplementary Fig. 6).

**Fig. 3:**
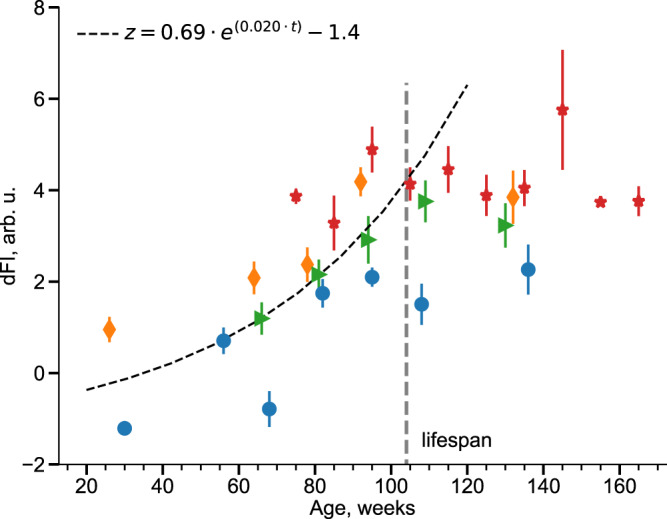
The dynamic frailty indicator (dFI) as a function of age. The dependence of the dFI from age in the validation datasets from the experiments: MA0071 (males, orange diamonds), MA0071 (females, blue circles), and MA0072 (green triangles). The black dashed line is the exponential fit in the age groups younger than the average lifespan of NIH Swiss mice (indicated by the vertical grey dashed line). Red stars mark the average dFI in age-matched groups of frail animals from the MA0073 cohort. All data are presented as Mean ± SEM.

dFI increased with the age of the animals both in the test and in the training sets (Fig. 3 and Supplementary Fig. 3, respectively). As expected from the qualitative solution of Eq. (1), dFI increased up to the age corresponding to the average animal lifespan (~100 weeks in our case). We performed an exponential fit in the form of Eq. (2) on the data from the test datasets (excluding animals that lived longer than the strain’s average lifespan and animals at the end of their life from the dataset MA0073). The calculation returned dFI growth exponent of *α* = 0.02 per week.

The saturation of the dFI beyond the average lifespan in the training and test datasets revealed a limiting value that is apparently incompatible with the animals’ survival. To highlight this possibility, we plotted the dFI ranges from a separate cohort of “unhealthy” mice from the MA0073 experiment, representing the animals scheduled for euthanasia under lab requirements (Fig. 3, red stars).

The character of the dFI acceleration with age and the dFI doubling rate matching the mortality acceleration rate (equal to 0.037 per week^22^) are good indicators of the association between dFI and mortality. The observation could be further supported by computing the Spearman’s rank correlation between the dFI “acceleration” (i.e., the difference between the dFI of an animal and its mean value in the corresponding age- and sex-matched cohorts) and the order of the death events among the animals of same age and sex (see Table 1). We obtained significant correlations between the dFI and the remaining lifespan for all cohorts.

**Table 1:** The dynamic frailty indicator (dFI) predicts the remaining lifespan

	M (26 w)	F (26 w)	M (52 w)	F (52 w)	M (78 w)	F (78 w)
**Cohort 1, animals**	91	125	148	188	173	150
**dFI**	**−0.31 (2.9e-03)**	**−0.18 (4.8e-02)**	**−0.38 (2.2e-06)**	**−0.23 (1.4e-03)**	**−0.37 (7.8e-07)**	**−0.28 (5.2e-04)**
**HR**_CBC_	**−0.31 (2.5e-03)**	−0.10 (2.6e-01)	**−0.41 (3.2e-07)**	**−0.20 (6.4e-03)**	**−0.37 (4.6e-07)**	**−0.35 (1.5e-05)**
**Cohort 2, animals**	79	118	139	177	133	129
**dFI**	**−0.34 (2.4e-03)**	**−0.21 (2.1e-02)**	**−0.37 (6.3e-06)**	**−0.25 (7.8e-04)**	**−0.32 (1.8e-04)**	**−0.31 (3.2e-04)**
**HR**_CBC_	**−0.31 (5.9e-03)**	−0.14 (1.2e-01)	**−0.40 (9.2e-07)**	**−0.22 (3.8e-03)**	**−0.31 (3.5e-04)**	**−0.39 (6.4e-06)**
**IGF1**	**−0.28 (7.7e-03)**	−0.17 (6.9e-02)	−0.12 (1.4e-01)	−0.11 (1.2e-01)	0.04 (6.9e-01)	0.05 (5.6e-01)
**Body weight**	**−0.26 (1.3e-02)**	**−0.19 (3.3e-02)**	**−0.25 (3.0e-03)**	**−0.24 (9.7e-04)**	0.03 (6.9e-01)	0.04 (6.8e-01)

We report Spearman’s correlation between the dFI and lifespan. The analysis is produced for the two cohorts: Cohort 1 includes all animals with mortality data; Cohort 2 includes the subset of animals from Cohort 1 for which insulin-like growth factor 1 (IGF1) measurements were available. For comparison, we characterize the performance of the Cox proportional hazard model (HR_CBC_) trained from complete blood counts in the same dataset. The values in brackets represent *p* values, a two-sided Spearman’s rank test. The significant correlations (*p* < 0.05) are highlighted in bold.

As a benchmark, we also produced a supervised Cox proportional hazards (PH) model predicting animal survival in the same data. Notably, the age- and sex-adjusted dFI predicted the remaining lifespan equally well or marginally better than the log-hazard ratio predictor from the supervised model trained in the same data (c.f. the rows corresponding to “dFI” and “HR_CBC_” in Table 1).

The dFI predicted remaining lifespan later in life better than body weight (BW) or insulin-like growth factor 1 (IGF1) serum level. Both factors were previously shown to be associated with mortality in refs. 23 and 24. As pointed out in ref. 24 and checked here, the concentration of IGF1 in serum was significantly associated with lifespan (*r* = − 0.28, *p* = 0.008) only in one cohort of younger, 26-week-old male mice. According to ref. 23 and our calculations, BW is better associated with mortality in the youngest animals (age 26 and 52 weeks).

### Late-life mortality deceleration and limiting mortality

The number of animals with recorded lifespans in MPD was insufficient to understand the deviations of mortality from Gompertz mortality law, as predicted by the aging at criticality model at the most advanced ages. Fortunately, a quantitative analysis of late-life mortality could be performed using the data from the non-treated controls in a large experiment involving thousands of mice^25^. Gompertz mortality fits produced the following estimations for the lifespan, $\bar{t}$, the Gompertz exponent, *α* and the initial mortality rate, *M*_0_ separately for males: $\bar{t}=100.3$ weeks, *α* = 0.0385 ± 0.0002 per week, *M*_0_ = (4.1 ± 0.1) ⋅ 10^−4^ per week; and for females: $\bar{t}=115.8$ weeks, *α* = 0.0568 ± 0.0003 per week, *M*_0_ = (3.4 ± 0.1) ⋅ 10^−5^ per week.

**Fig. 4:**
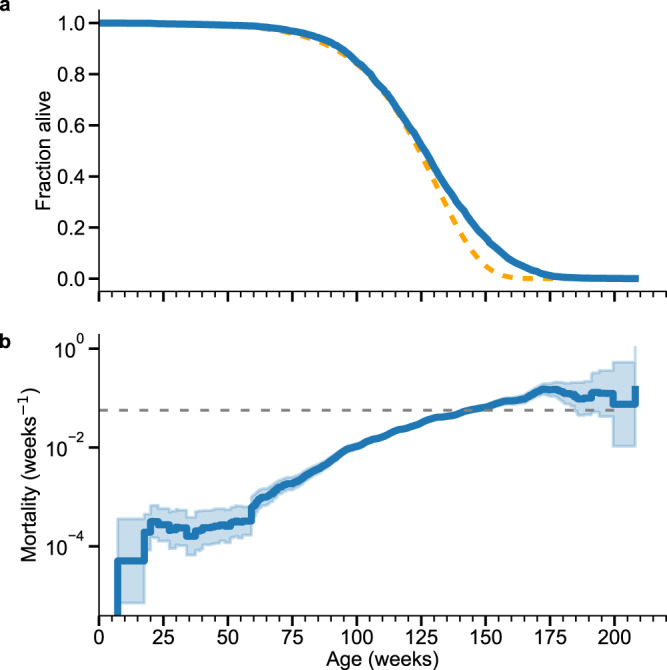
Late-life mortality deceleration in mice. **a** The Kaplan-Meier survival curve (solid blue line) in female mice cohorts from ref. 25. The orange dashed line represent the best Gompertz fits. **b** The Nelson–Aalen estimator of total mortality (solid blue line) and the 95% confidence intervals are filled in blue. The grey dashed line corresponds to the mortality level according to the theoretical prediction $M(t\, \gg \,\bar{t})=\alpha$.

Late in life in both sexes, the empirical survival curves fall slower than the predictions from the Gompertz mortality law (see the solid blue and the orange dashed lines in Fig. 4a and Supplementary Fig. 4a, respectively). This is a signature of the deceleration of mortality. The blue lines in Fig. 4b and Supplementary Fig. 4b are the empirical mortality curves. The grey dashed lines correspond to the theoretical expectation for the level of mortality at the plateau, corresponding to the theoretical limiting mortality $M(t\, \gg \,\bar{t})=\alpha$, where *α* is the mortality rate doubling rate from the Gompertz fit.

The leveling off of mortality is better pronounced in the female cohorts (Fig. 4) and is consistent with the theoretical prediction of the late-life plateau mortality in our model. In the male cohorts, the animals expire before the plateau is reached (Supplementary Fig. 4), possibly due to the elevated background mortality earlier in life between weeks 40 and 100. Nevertheless, the maximum observed mortality is still not very far from (and is definitely of the same order as) the predicted limiting value even in male cohorts.

### dFI and hallmarks of aging

**Fig. 5:**
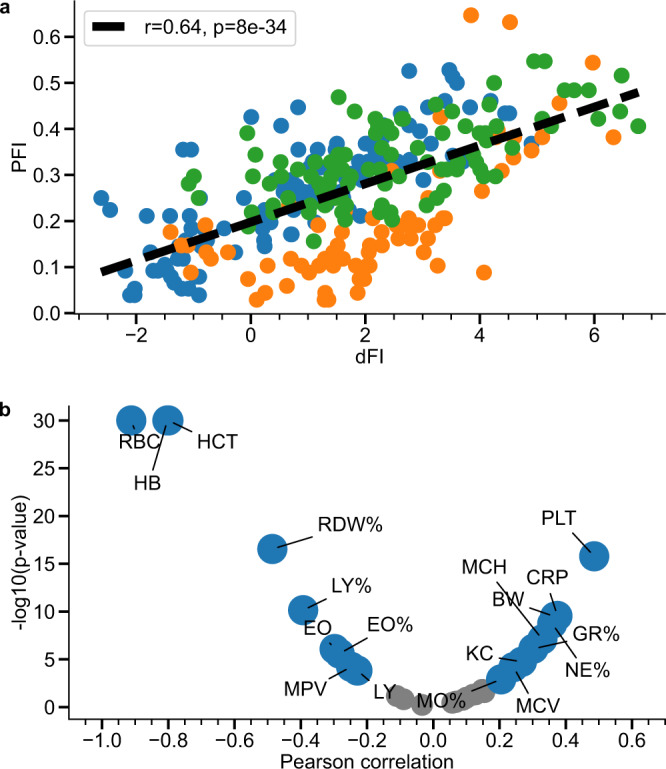
Correlation of dFI with other biomarkers of aging. **a** Correlation between the dFI and the physiological frailty index (PFI). Colors represent animals from the test dataset, where blue, orange and green circles are females in MA0071, males in MA0071 and males in MA0072, respectively. **b** Volcano plot representation of the dFI correlation with the extended set of biomarkers in the test datasets MA0071 and MA0072. Features with correlation above and below significance level *p* < 0.001 are shown with grey and blue circles, respectively. The most significant correlations (excluded dFI components) were between dFI and C-reactive protein (CRP), red cell distribution width (RDW), body weight (BW), and murine chemokine CXCL1 (KC). Two-sided *p* values were calculated for the Pearson correlation coefficient and the sample size of *n* = 282 animals.

To further validate dFI as a biomarker of aging, we examined its relation to the PFI, a quantitative measure of aging and frailty recently proposed in ref. 3. dFI and PFI were found to be strongly correlated (Pearson’s *r* = 0.64, *p* < 0.001), Fig. 5a. PFI is a composite frailty score and also depends on CBC measures for its determination. However, the calculation of PFI involves more factors, including traditional measures of frailty in animals and humans, such as grip strength, cardiovascular health, inflammation markers, etc. Remarkably, the correlation between PFI and dFI remained significant after adjustment for sex and age (Supplementary Fig. 5, Pearson’s *r* = 0.59, *p* < 0.001).

As illustrated in Fig. 5b and Supplementary Fig. 6, 7, we observed that the dFI was significantly associated with an extended set of CBC features across independent functional subsystems (most notably, but not limited to, myeloid cell lineage). The correlation between dFI and myeloid cell features was less profound in the training set, involving multiple strains (Supplementary Fig. 7). The correlation coefficient is a measure of the response of dFI to individual CBC features variation and is different (sometimes even of opposite sign) in various mouse strains (Supplementary Fig. 8). The variation of the associations of individual features and dFI would be a significant challenge to any linear model and is a demonstration of the non-linear character of the AE.

The dFI was strongly associated with red blood cell distribution width (RDW) and BW (Fig. 5b), known predictors of frailty in both mice^26^ and humans^27^. dFI was also strongly associated with levels of C-reactive protein (CRP, *r* = 0.39, *p* < 0.001) and the murine chemokine CXCL1 (KC, *r* = 0.28, *p* < 0.001), both of which are known markers of systemic inflammation and mortality^28,29^.

**Fig. 6:**
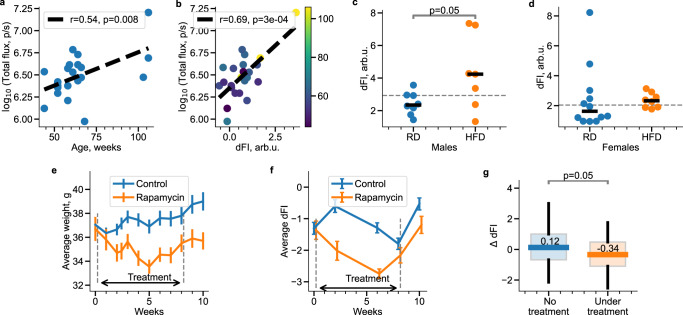
dFI is associated with senescent cells burden and responds to lifespan modulating interventions. Total flux (TF) in log scale represents p16-dependent luciferase reporter activity, is a quantitative indicator of senescent cells burden, and shows statistically significant correlations with age (Pearson’s *r* = 0.54, two-sided *p* = 0.008, *n* = 23 animals) (**a**) and with dFI (Pearson’s *r* = 0.69, two-sided *p* = 0.0003, *n* = 23 animals) (**b**) in old mice (>50 weeks). The colorbar in **b** represents animal age in weeks. dFI responds to the lifespan-modifying effect of high-fat diet (HFD): the dFI values (dots) were obtained late in life (at week 78) for male (**c**) and female (**d**) mice fed with RD or HFD. The horizontal bar and the dashed lines indicate the mean values in the groups and for all animals, respectively. dFI was significantly higher in males with HFD (*n* = 7 animals) vs RD (two-sided *p* = 0.05, Student’s *t* test, *n* = 8 animals), but there was no significant difference between HFD (*n* = 8 animals) and RD (*n* = 12 animals) groups of female mice. The dFI measurements are consistent with life-shortening and neutral effects of HFD in males and female animals in the same experiment, as reported in ref. 3. Effects of 8 week-long rapamycin treatment on body weight and dFI. Body weight (**e**) and dFI (**f**) were measured every 1 and 2 weeks, respectively. All the data are presented as the mean ± SEM (*n* = 48 and *n* = 12 mice in control and rapamycin groups, respectively). **g** The statistics of the dFI increments in pairs of consecutive measurements are different by the presence or the absence of the treatment (the blue box, including both the control and rapamycin-treated group after cessation of the treatment, *n* = 204 animals, vs. the treated group, the orange box, *n* = 36 animals). Boxplots indicate median, 25th and 75th percentiles, whiskers indicate 5th and 95th percentiles. The dFI increments between the subsequent measurements were significantly lower under treatment (*p* = 0.05, Student’s two-tailed *t* test), thus suggesting lifespan increasing effects of rapamycin.

Aging is associated with an increasing burden of senescent cells^30–32^, widely considered a source of chronic sterile systemic inflammation, “inflammaging”^33^. Senescent cells (SC) are commonly detected in vivo as a population of p16/Ink4a-positive cells accumulated with age recognized by the activity of p16/Ink4a promoter-driven reporters^34^. We utilized homozygous p16/Ink4a reporter mice with one p16/Ink4a allele knocked in with firefly luciferase cDNA^35^. Figure 6a shows the correlation between animal age and the presence of senescent cells, as measured by the flux from p16/Ink4a promoter-driven luciferase activity (*r* = 0.54, *p* = 0.008).

The correlation of this SC proxy (total luciferase flux) with dFI was even stronger (*r* = 0.69, *p* < 0.001; Fig. 6b). The observation fits well with our theoretical prediction: the kinetic equation for the order parameter (dFI) is autonomous (its coefficients do not depend on the time). This means that the organism state variables, including measures of senescent cell burden, may not explicitly depend on age, so the age dependence may only come through the dependence on dFI.

### dFI responds to lifespan-modulating interventions

To assess the utility of dFI for the analysis of effects of interventions on aging, we retrospectively evaluated the dFI and its relation to the remaining lifespan in response to a high-fat diet (HFD) using the data from the earlier experiment^3^. Male mice were fed HFD instead of a regular diet (RD) beginning at 50 weeks of age had significantly reduced lifespans (Supplementary Fig. 9a) and also showed a significant increase in average dFI measured at week 78 (*p* = 0.05, Student’s two-tailed t-test; Fig. 6c) in comparison to control RD-fed males. In contrast, HFD feeding of female mice had no effect on either lifespan or average dFI (Supplementary Fig. 9b and Fig. 6d). Thus, dFI appeared to be a good predictor of gender-dependent differences in the organism to HFD, the underlying reasons for which remain to be explained.

Our calculations shown that the aging acceleration captured by the dFI agreed with the previously reported gain in the PFI (*p* = 0.02 according to Student’s t-test) in male and no effect in female mice at week 78 in the same experiment. This is a notable statement regarding the sensitivity of dFI, since PFI was developed (trained) in the MA0071 experiment and employs more (18 vs. 12) variables than mere CBC for its determination. On the contrary, we did not use any data from the MA0071-73 datasets to train the dFI model. Figure 6c, d demonstrates dFI performance relative to the independently developed PFI in an external dataset.

In another experiment, we tested the response of dFI to a short treatment with an established life-extending agent, rapamycin^36,37^. Here we present the results of an experiment with a cohort of 60 60-week-old C57BL/6 male mice. 48 animals (the treatment group) were treated with rapamycin daily at a dose of 12 mg/kg for 8 weeks. The other 12 animals (the control group) received the vehicle on the same schedule.

BWs were measured every week and increased as expected in the control group (Fig. 6e). In contrast, BW in the rapamycin-treated group stayed approximately constant near the initial value throughout the observation period of 10 weeks. A lower BW is typical for rapamycin-treated mice compared to the control group^3,38^. To generate dFI values for the mice in this experiment, we collected and produced CBC from blood samples from each animal every two weeks (Fig. 6f).

The longitudinal character of sampling in the experiment let us use the autoregression analysis to detect possible effects of a drug on the dynamics of dFI and hence aging. Whenever a non-random force (that is the effect of the drug) is present in Eq. (1), the jump in dFI between any of the consequent measurements from the same animal should satisfy the modified Eq. (7):

4 $$z(t+\Delta t)=rz(t)+{z}^{\prime}+J+\xi,$$

where *J* is the accumulated effect of the drug along the aging trajectory between the measurements. The time intervals are usually very small, *α*Δ*t* ≪ 1 and hence the autoregression coefficient *r* ≈ 1. We, therefore, expected to identify the effect of rapamycin by comparing the distributions of the dFI increments, ΔdFI = *z*(*t* + Δ*t*) − *z*(*t*), in animals receiving or not the treatment between the measurements both in the treated and in the control groups (see Methods for the details of the statistical analysis). The statistical analysis of the dFI increments demonstrated a significant aging deceleration associated with the rapamycin treatment (Fig. 6g, *p* = 0.05, Student’s two-tailed test).

## Discussion

We introduced a novel way to reveal biomarkers of age and frailty from big biomedical data involving longitudinal measurements, i.e., multiple samples of the same animals collected along the aging trajectories. The approach belongs to the class of unsupervised learning algorithms such that it does not require labels associated with age, mortality, and morbidity. It could be exemplified by the discovery and characterization of a biomarker of aging in mice, the dFI, from conventional and automated measurements of CBC and trained from the data from MPD.

Aging is a very slow process that occurs at characteristic time scales far exceeding times associated with molecular processes or operations of an organism’s functional subsystems. Typically, such a hierarchy of scales arises from criticality, which is a special case of a dynamic system operating close to a tipping point separating the stable and unstable region^15–17,39^. In ref. 18, we proposed that aging results from inherent dynamic instability of the underlying regulatory networks and manifests itself as small deviations of the organism state variables (physiological indices) get exponentially amplified and lead to the exponential acceleration of mortality. The first principal component score is then an approximation to the order parameter characterizing the unstable phase and having the meaning of the total number of regulatory errors accumulated in the course of life of the animal^17^. Hence, we believe that aging at criticality conjecture provides a good explanation for the success of Principal Components Analysis (PCA) as a semi-quantitative tool in aging research^40–42^.

The idea of the order parameter associated with instability is a generalization of a concept initially introduced in the Ginzburg-Landau theory to describe phase transitions in thermodynamics. The idea was further developed for applications to open non-equilibrium systems in the form of the “enslaving-principle”^13^, which states that next to the critical point, the dynamics of fast-relaxing (stable) components of a system is completely determined by the ’slow’ dynamics of only a few ’order-parameters’. The dFI identified as an approximation to the order parameter is then not a mere machine learning tool developed for specific predictions but rather a fundamental macroscopic property of the aging organism as a non-equilibrium system.

However, the abilities of linear rank reduction techniques, such as PCA, to unravel an accurate dynamic description of aging are limited for the following reasons. First, there are no reasons to believe that the effects of non-linear interactions between different dynamic subsystems are small. That is why a biomarker produced from such a linear analysis cannot be expected to perform well in different biological contexts (strains, laboratory conditions, or therapeutic interventions such as drugs). Second, biological measurements are often noisy. Hence, simple techniques lacking efficient regularization may fail to reconstruct the space of the latent variables correctly unless a prohibitively large number of samples is obtained^43^. Finally, the association of the first principal component with the order parameter is only an approximate statement. Fundamentally, there is no way to identify the system’s dynamics from the data, which does not include the dynamics itself in multiple measurements of the same organism along the aging trajectory.

To compensate for the drawbacks of PCA and demonstrate an efficient way to produce an accurate approximation to dFI from the data, we employed an artificial neural network, a combination of a deep denoising AE and an AR model. The AE part of the algorithm is a non-linear generalization of PCA and was used to compress the correlated and necessarily noisy biological measurements into a compact set of latent variables, a low-dimensional representation of the organism state. The AR-arm of the network is nothing else but the best possible prediction of the future state of the same animal from the current measurements. The solution to the AR-problem provided by the network is the estimate for the order parameter from the given data, the dynamic frailty indicator (dFI).

The network architecture applied here was inspired by deep rank-reduction architectures, recently used to characterize numerical solutions of large non-linear dynamical systems^44,45^. In a broader sense, we report the development and characterization of a fully interpretable (model-based) algorithm, a particular example of identifying the equations of motion of the underlying dynamical system from data with the help of deep learning^46,47^. By the standard of physical sciences, aging in mice turns out to be “exactly solvable”. We demonstrated an analytically tractable model tying the dynamics of the physiological state and all-cause survival to the evolution of a single age-dependent quantity, the order parameter of the unstable phase, and following a simple first-order stochastic differential equation.

The profound correlations between the CBC features and dFI may reflect a key role in aging of hematopoietic tissue in determining aging of the whole organism. This concept is intuitively acceptable given the universal systemic physiological function of blood. As an alternative explanation, age-dependent changes in blood parameters may be secondary events induced by aging of the remainder of the organism (i.e., various solid tissues). However, accumulated experimental evidence argues against this. Multiple reports are demonstrating “rejuvenating” effects of young hematopoietic system on old animals delivered either by bone marrow transplantation or by parabiosis (reviewed in ref. 48). Moreover, restoration of mouse hematopoiesis through transplantation of hematopoietic stem cells (HSCs) from young vs. old donors clearly demonstrated that aged HSCs cannot be rejuvenated by the environment of a young body^49^. Also, the interpretation of age dependence of HSC-derived features as secondary effects of aging would face formal difficulties since the dynamics of such factors should exhibit shorter, in fact at least twice shorter, doubling times than the dFI and the mortality rate doubling times.

A peculiar result of our analysis is that our data strongly point toward myeloid lineage which provides much more accurate predictors of biological age than lymphoid lineage parameters. This is counterintuitive since aging is generally accepted to be associated with the well-documented general decline in immunity known as an immunosenescence^50^, the phenomenon illustrated by the reduced efficiency of vaccination^51^ and increased frequency and lethality of infectious diseases and cancer in older organisms^52^. Nevertheless, there is strong experimental evidence that supports and provides a mechanistic explanation for our finding that myeloid parameters weigh more heavily than lymphoid ones as biological age indicators. In a comprehensive study of the epigenetic mechanisms of HSC aging, Beerman et al.^49^ described age-dependent epigenetic reprogramming that leads to a significant shift towards myeloid lineage differentiation of the progeny of aged HSCs^49,53^. This shift is driven by specific changes in methylation of the DNA of HSCs that occur during mouse aging. Surprisingly, these changes in methylation, which alter gene expression, do not occur in the part of the genome that controls HSC phenotype, but rather modify DNA regions encoding genes that control downstream differentiation stages. Remarkably, the pattern of DNA methylation changes associated with aging of HSCs seems to represent the same process that was previously described as a DNA methylation-based clock^1,49^, and therefore, may be part of the same epigenetically controlled fundamental aging mechanism. Another factor that could diminish the impact of lymphoid lineage-related parameters as biological age markers is the reactive nature of this branch of hematopoiesis, which serves to rapidly respond to sporadic events such as viral or bacterial infection, wounding, and other types of stress requiring an emergency response usually in the form of acute inflammation. Since the time of occurrence of such events is unpredictable, age-associated changes may be masked by the noise coming from large-scale age-unrelated fluctuations in the lymphoid compartment.

These observations do not mean that the blood is the single determinant of aging (otherwise, biological age would be 100% defined by the age of HSCs), but at least place it among the major drivers of the process and provide an explanation for our success in reliably determining biological age from blood test data. Aging in our model emerges cooperatively so that despite the appearance of “aging clock” in the form of the order parameter or its estimate from the data, the dFI, there is no specific subsystem tracking time (or the age) in an animal.

The organism state’s dynamics are described by an autonomous Eq. (1), where none of the model coefficients explicitly depend on time. Accordingly, no physiological state variable can depend on the age of the animals explicitly, only implicitly via dependence on the collective variable, the dFI. That is why we observed the correlations between the dFI, on the one hand, and multiple aging and frailty measures commonly known as the hallmarks of aging^54^, on the other. These include grip strength, BW, RDW, and markers of inflammation such as CRP and KC (IL-8). dFI also correlated well with the p16-luciferase flux, a proxy for the number of senescent cells in aged mice. Conversely, we predict that anti-aging treatment altering the dFI should significantly alleviate other hallmarks of aging at the same time. This property is not uncommon among interventions with potentially life-extending effect^55^.

Moreover, the instability of the organism state means that the animals cannot relax to any equilibrium even after a short perturbation (formally, this property manifests itself as strong auto-correlations of dFI over extended periods of time). Therefore, the effects of short treatments should likely persist until the end of life. Accordingly, the effects of such transient treatments could, in principle, be detected in short experiments involving longitudinal dFI measurements in just over a few weeks. Although the effect size and the signal-to-noise ratio in the short prospective rapamycin treatment experiment are not very large, the aging deceleration observed in this work is compatible with the earlier reported life-long pro-longevity effects of transient rapamycin treatments^37^.

The common biomarkers of aging rely on individual or composite measures of the deviation of an organism from its youthful state. Notable examples are forms of frailty index (FI) defined as the proportion of accumulated deficits in any available signal^8,9^ or, more specifically in blood test data^9–11,56,57^. Since we do not expect any dependence of the physiological state variables on age other than the dependence on the order parameter, there should be a good concordance between composite markers of aging and dFI. Indeed, we observed a very high degree of concordance between the dFI and the PFI from ref. 3, which is an example of the Mahalanobis distance from old to young states^7^ and is derived from a wide range of features including CBC, physical fitness, cardiovascular health, and biochemistry.

Of all such measures, dFI alone comes with an established equation of motion (1) and hence has the best auto-correlation property along the individual aging trajectories (see, e.g., the auto-correlation of dFI and the first PC in Fig. 2 and Supplementary Fig. 2, respectively). The property is convenient for determining the effects of drugs or other interventions on aging in experiments involving comparisons of the organism state of the same animals before and during the treatments.

The reduction of aging to the dynamics of a single variable makes the quantitative model of aging in the form of the stochastic Eq. (1) from ref. 18 and this study distinct from previous proposals to derive mortality from a physiological state’s dynamics^58,59^. Such models are mathematical metaphors of random walks in very high dimensional spaces and thus may be difficult to interpret or infer from biological signals without additional assumptions. We believe that criticality is a helpful theoretical observation and deep learning is a great practical tool, which may be used together to simplify identifying the model parameters and their relations to organism properties. We note that the explanation of the Gompertz law of mortality is insufficient proof of an aging theory. Matching stochastic longitudinal dynamics of physiological indices to a model prediction is a much harder challenge and should be used as an effective tool for model validation.

dFI increased exponentially with age at a characteristic doubling rate of 0.02 per week. This estimate is somewhat smaller than (but still of the same order as) the expected Gompertz mortality acceleration rate of 0.037 per week^22^ for the SWR/J strain. Given the observed dFI doubling rates in mice, $\alpha \bar{t} \sim 3$ and hence the deterministic phenotypic changes dominate the random effects by a factor of 3 (see Eq. (2) and Fig. 3).

More interestingly, in the cross-sectional dataset, the dFI saturated at a limiting value, which is reached at the age corresponding to the average lifespan in the group. However, we observed that the dFI ceiling corresponds to the dFI levels in cohorts of animals scheduled for euthanasia due to excessive morbidity under current laboratory protocols, which is as close to death from natural causes as animals could possibly be in a modern laboratory.

Both features of the aging trajectories in MPD are compatible with the analytical solutions of Eq. (1) for the dynamics of the order parameter. In ref. 18, we explained that early in life dFI increases exponentially (see Eq. (2)). At the age approximately corresponding to the average lifespan in the population, non-linear effects take over the dynamics of dFI, and the organism state deviates from its youthful state even faster than exponentially. Such a situation is incompatible with survival and hence cannot be observed in the data. In our model and in the experiment, death occurs quickly once the maximum dFI level is reached at some point in the life history of the animal.

The stochastic Eq. (1) establishes the “law of motion” for the organism’s physiological state and predicts the late-life mortality deceleration in the form of saturation of mortality at the plateau level, $M(t\, \gg \,\bar{t})\, \approx \,\alpha$. The predicted relationship between the limiting mortality and the mortality rate doubling time held in a variety of species^60^ and experiments in multiple conditions in the same species, such as nematodes^42^. Here we report the validation of the limiting mortality prediction in very large cohorts of mice from ref. 25.

The good semi-quantitative agreement between the empirical mortality curves in large experiments and the theoretical prediction provides an independent and sensitive test of the aging at criticality model as a theoretical framework proposed here for the data analysis in experiments involving aging animals. More specifically, the experimental confirmation for the late-life mortality deceleration prediction validates the basic stochastic Eq. (1) and the association between its solution in the form of dFI and all-cause mortality. We also note that the mortality deceleration in the model arises from the stochastic nature of the order parameter dynamics and should be expected even in a cohort of genetically identical animals (see the discussion in ref. 61).

Deviations from the Gompertz law in human cohorts also occur, but at ages exceeding the average lifespan when the mortality is already well beyond the theoretical limit corresponding to the mortality rate doubling rate by almost an order of magnitude^60^. This means that the character of the organism state dynamics in the course of human aging is qualitatively different than that in mice or nematodes. In ref. 5, we observed that the fluctuations of physiological indices in humans are also dominated by a collective variable characterized by a relatively long but finite auto-correlation time (in the range of a few weeks) and associated with age and all-cause mortality. The number of individuals exhibiting signs of the loss of dynamic stability (measured by exceedingly long auto-correlation times) increased exponentially with age at a rate matching the mortality doubling rate from the Gompertz mortality law^61^.

The intimate relation between the auto-correlation properties of physiological state variables and hallmarks of aging suggests that AR analysis enhanced by deep learning may help discover signatures of human aging and chronic disease progression. The benefit may be particularly huge in studies involving large sets of longitudinal measurements but often lacking follow-up mortality and morbidity information. While hallmarks of aging in mice are correlated and primarily reversible, a large part of physiological changes associated with aging in humans is stochastic and may be thermodynamically irreversible^62^. Therefore, we expect that the systematic application of dynamic systems theory principles to biomedical data analysis will help identify actionable aging phenotypes and thus facilitate the discovery and development of anti-aging therapeutics that produce lasting rejuvenating effects.

## Methods

### Datasets

The training data set was prepared from the nine data sources available in the MPD^19^. A list of the included sources is presented in Supplementary Data 1 with references to the included and missing records grouped by sex and age cohorts. We used the assays providing the CBC data only, assays with other biomarkers were not considered due to the insufficient number of samples. Our model was trained using the best overlap of available CBC features from all sources. The final list contained 12 CBC features: granulocytes differential (GR%), granulocytes count (GR), hemoglobin (HB), hematocrit (HCT%), lymphocyte differential (LY%), lymphocyte count (LY), mean corpuscular hemoglobin content (MCHC), mean hemoglobin concentration (MCH), mean corpuscular volume (MCV), platelet count (PLT), red blood cell count (RBC) and white blood cell count (WBC). See Supplementary Data 2 for the list of all abbreviations. If the data source lacked granulocytes measurements, it was retrieved using formulas:

5 $${{{{{{{\rm{GR}}}}}}}}	={{{{{{{\rm{WBC}}}}}}}} - {{{{{{{\rm{LY}}}}}}}} - {{{{{{{\rm{MO}}}}}}}}\\ {{{{{{{\rm{GR}}}}}}}}\%	=100 - {{{{{{{\rm{LY}}}}}}}}\%-{{{{{{{\rm{MO}}}}}}}}\%$$

All animals with one or more missing parameters were excluded from the training. The percentage of the excluded records was <2% and should not have affected the results.

### Animals

All animal experiments were approved by the Institutional Animal Care and Use Committee of Roswell Park Cancer Institute or by the Explora BioLabs, Inc Animal Use Committee.

We received 4–5-week-old NIH Swiss male and female mice from Charles River Laboratories (Wilmington, MA). They were allowed to age within the Roswell Park Comprehensive Cancer Center (RPCCC) animal facility. During this time mice were housed 1–3 per cage and were fed ad lib with standard chow (Tekland Global 18% Protein Rodent Diet). Blood samples were obtained at different ages as part of creating of the PFI^3^. Blood samples were collected from a single submandibular vein bleed into EDTA-treated Vacutainer tubes (total volume of 20 *μ*l) and used for whole blood cell counts and glucose measurements using Hemavet 950 Analyzer (Drew Scientific). Another 75 *μ*l of blood was collected into Li-Heparin treated plasma separator tubes; plasma was purified by centrifugation at 5000×*g* for 5 min and used for measuring the concentration of circulating pro-inflammatory cytokines and triglycerides.

Dataset MA0071 was built in a cross-sectional experiment using male and female NIH Swiss mice. Blood was collected from male mice at ages of 26 (*n* = 20), 64 (*n* = 18), 78 (*n* = 17), 92 (*n* = 14), and 132 (*n* = 6) weeks. Female age groups were represented by the ages of 30 (*n* = 20), 56 (*n* = 20), 68 (*n* = 19), 82 (*n* = 19), 95 (*n* = 18), 108 (*n* = 20), and 136 (*n* = 7) weeks. Dataset MA0072 was obtained from a longitudinal experiment. Blood samples were collected at the ages of 66 (*n* = 27), 81 (*n* = 22), 94 (*n* = 21), 109 (*n* = 16), and 130 (*n* = 7) weeks. Dataset MA0073 includes blood samples collected from 97 male and 127 female mice of different ages when animals reached approved experimental endpoints and required humane euthanasia. All animal procedures were performed according to the approved Institutional Animal Care and Use Committee (IACUC) protocol. Mice were monitored daily for the development of age-related pathologies. Whenever health issues were reported, research staff contacted veterinarian staff and followed their recommendations for either treatment or euthanasia. Mice were treated until the condition was improved or euthanized when the endpoint for each health condition described in the protocol was reached. Euthanasia was performed by CO2 asphyxiation followed by cervical dislocation. See Supplementary Data 3 for the total number of animals in these datasets.

p16/INK4a-LUC female mice (p16-Luc) at ages of 44 to 106 weeks were obtained from the N. Sharpless laboratory at the University of North Carolina (Chapel Hill, NC). All animals were housed under 12:12 light:dark conditions (12 hours of light followed by 12 hours of darkness) at the Laboratory Animal Shared Resource at RPCCC. All animal experiments were approved by the IACUC of Roswell Park Cancer Institute. Bioluminescence imaging was performed using an IVIS Spectrum imaging system (Caliper LifeSciences, Inc, Waltham, MA). p16/Ink4a-Luc+/- mice were injected intraperitoneally with D-Luciferin (150 mg/kg, Gold Biotechnology), 3 minutes later anesthetized with isoflurane and imaged using a 20-second integration time and medium binning. The images were processed and quantified as the sum of photon flux recorded from both sides of each mouse using Living Image software (Perkin Elmer, Waltham, MA.).

For the rapamycin treatment experiment 60-week-old C57BL/6J male mice were obtained from Jackson Laboratories (USA). The cohort of 60 60-week-old C57BL/6 male mice was divided into treatment (*n* = 12) and control (*n* = 48) groups using a stratified randomization technique to produce indistinguishable distributions of dFI values prior to the experiment. The blood samples (total volume of 120 *μ*L) were collected into EDTA tubes via submandibular or facial vein using a lancet. All animal procedures were approved by the Explora BioLabs, Inc animal use committee (IACUC SP17-004-035B) and were in accordance with Explora BioLabs, Inc policies on the care, welfare, and treatment of laboratory animals. Rapamycin was purchased from LC Laboratories (MA, USA). Rapamycin was administered daily at 12 mg/kg via oral gavage for 8 weeks. The control group was treated with vehicle (5% Tween-80, 5% PEG-400, 3% DMSO).

### Dimensionality reduction with PCA

We performed the PCA with the help of Python and Scikit-learn package^63^. First, we applied PCA transformation to the entire training dataset. However, the principal components were dominated by the difference in mice strains. We removed strain difference by subtracting mean values of CBC features calculated for the earliest age available for the selected strain from values of CBC features of all animals for this strain:

6 $$\widetilde{{X}_{i}^{j}}={X}_{i}^{j}-\frac{1}{{N}_{t}}\mathop{\sum }\limits_{t\!=\!\min ({{{{{{{\rm{age}}}}}}}})}^{{N}_{t}}{X}_{i,t}^{j},$$

where the indices *i* and *j* enumerate the CBC features and strains, respectively, and *t* is the age of an animal. For simplicity, we filtered out mouse strains, which were not presented in the Peters4 dataset^64^.

Most of the variance in the data representing the full dataset was associated with animal growth and maturation. The first PC score increased with the age of animals most notably after 25 weeks (Supplementary Fig. 1). On the contrary, the second and the third PC scores acquire non-zero means by the same age of 25 weeks. This suggests that aging and early development in mice are different phenotypes. Subsequently, we performed all our calculations using the data from animals older than 25 weeks.

### Statistical analysis of mortality data

The death records for animals linked with the MPD dataset Peters4 were also available in the MPD as a separate dataset named Yuan2^23^. These datasets contain different cohorts of animals with a vast overlap. We found mortality records for 487 animals in Peters4 dataset, while 393 animals were missing. The reason for the missingness is unknown. To create censoring records, we included all animals having at least two sequential CBC measurements. We assumed that the animals provided with a single CBC measurement were probably sacrificed after the blood collection. Hence, if an animal has more than one CBC measurement, we consider it lost at the last measurement. Altogether, we found 79 animals satisfying this condition. The rest 314 animals with unknown mortality dates were excluded from the analysis.

The Spearman’s rank correlation test was performed separately for the two cohorts of mice. The first cohort included all animals from the Peters4 dataset with uncensored mortality records. The second cohort included animals from the Peters4 dataset with the measurements of BW and the IGF1 serum level taken from the MPD dataset named Yuan1^24^.

We performed the Cox PH regression analysis with the help of Python and Lifelines package^65^. First, we produced the supervised multivariate Cox-PH model using the age, sex, and CBC features as covariates and utilizing the data from all the animals from the Peters4 dataset, including both censored and uncensored mortality events. The output of the model, the log-hazard ratio (HR_CBC_) is the dot product of the vector comprising the CBC features and the respective coefficients from the Cox-PH model. Next, we tested the association of the HR_CBC_ feature in a univariate Cox-PH model alongside the dFI score in cohorts of animals of the same age and sex (see Supplementary Data 6) as an alternative for Spearman’s rank correlation test. Both tests were in good agreement with each other.

### Training of the AE/AR neural network

We used a combination of a deep AE and a simple AR model for modal analysis (AE-AR model). At its bottleneck, the encoder arm of the AE produced a compressed 4-dimensional representation **y** of the input, the 12-dimensional physiological state vectors **x** built from the available CBC measurements. The decoder arm reconstructed the original 12-dimensional state $\tilde{{{{{{{{\bf{x}}}}}}}}}$ from the bottleneck features.

Simultaneously with the AE, we trained the network to fit the longitudinal slice of MPD (including fully-grown animals at ages from 26 to 104 weeks with a sampling interval of Δ*t* = 26 weeks) to the solution of the linearized (*g* = 0) version of Eq. (1),

7 $$z(t+\Delta t)=rz(t)+{z}^{\prime}+\xi,$$

where *z* is the best possible linear combination of AE bottle-neck features, $r=\exp (\alpha \Delta t)\, \approx \,1$, and ${z}^{\prime}$ are the best fit values of the auto-regression coefficient and the constant shift, respectively. Here, *ξ* is the error of the fit (the combination of the system’s noise and measurement errors).

**Fig. 7:**
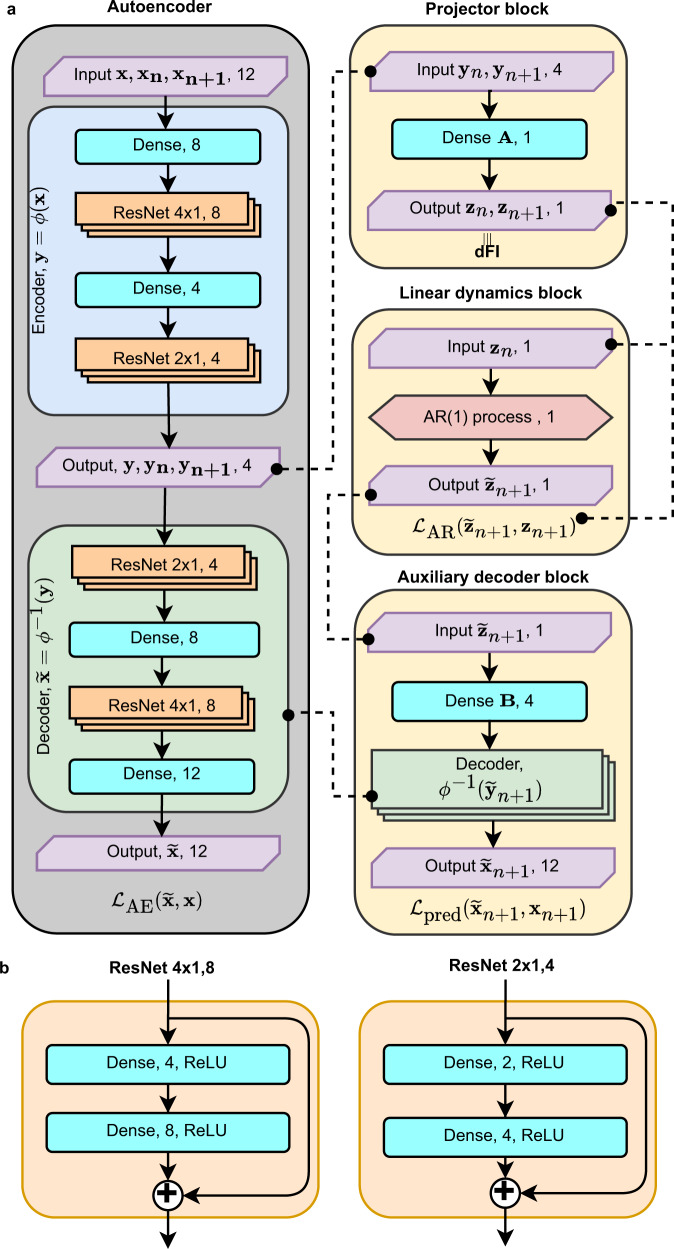
The schematic representation of the network architecture used to train dFI. **a** The network includes of nonlinear auto-encoder (AE) and the auto-regression model (AR), the linear projector, and the decoder blocks. The output of the projector block was designated as dFI. **b** Schematic representations of the residual network (ResNet) blocks.

We adapted the neural network architecture proposed in ref. 45. Our implementation handled cross-sectional and longitudinal measurements simultaneously with an imbalance in favor of cross-sectional data, which is typical for real-world clinical data. As inputs, the network has three 12-dimensional vectors represented by CBC parameters: one for the cross-sectional dataset (**x**), and two others for the longitudinal dataset corresponding to the present (**x**_*n*_) and future (**x**_*n*+1_) states of a sample (Fig. 7a).

The cross-sectional samples served as inputs in the AE. The longitudinal samples in the compressed representation (**y**_*n*_, **y**_*n*+1_) also participated in the training autoregression part. The main advantage of the inclusion of AE in the neural network is its ability of effective nonlinear dimensionality reduction^66^, which is necessary for such correlated quantities as CBC components (Fig. 1a). The reduced size of latent dimensions works as regularization and helps to train without overfitting on a small longitudinal dataset using more samples from a larger cross-sectional dataset.

The AE block encodes input (**x**) to the 4-dimensional vector **y** = *ϕ*(**x**) and then reconstructs back the original signal $\widetilde{{{{{{{{\bf{x}}}}}}}}}={\phi }^{-1}({{{{{{{\bf{y}}}}}}}})$. AE was implemented as a stack of fully connected dense layers and residual network blocks (ResNet)^67^. The dense layers have a trainable weight matrix **W**, bias vector **b**, and a linear activation function by default. The ResNet block, shown in Fig. 7b, is a stack of two dense layers with an activation function of a leaky rectified linear unit (Leaky ReLU). The input and the output are linked by applying element-wise addition. The ResNet blocks add nonlinear rectification transformations to the original input, helping to learn nonlinear transformations. The AE is trained simultaneously on cross-sectional and longitudinal datasets.

The projector block takes a 4-dimensional vector as an input and transforms it to a scalar *z* = **A** ⋅ **y**, which we refer to as dFI. During training, a pair of vectors is fed to the inputs: one *y*_*n*_ for the present state of the system and one *y*_*n*+1_ for the future state. The linear dynamics block solves the autoregression problem (7) and predicts the future state ${\widetilde{z}}_{n+1}=\xi ({z}_{n})=r{z}_{n}+b$. The auxiliary decoder block reconstructs the original 12-dimension CBC vector from the output of the linear dynamics block ${\widetilde{z}}_{n+1}$ utilizing the decoder *ϕ*^−1^ from the AE block: ${\widetilde{{{{{{{{\bf{x}}}}}}}}}}_{n+1}={\phi }^{-1}({{{{{{{\bf{B}}}}}}}}\cdot {\widetilde{{{{{{{{\bf{z}}}}}}}}}}_{n+1})$.

To force matrices of **A** and **B** in the projector and linear dynamics blocks to be left and right eigenvectors in the solution of Eq. (7) we added the following constraints:

8 $$\begin{array}{l}{C}_{AB}:\, \parallel {{{{{{{\bf{A}}}}}}}}\cdot {{{{{{{\bf{B}}}}}}}}-{{{{{{{\bf{I}}}}}}}}{\parallel }_{F}^{2}=0\\ {C}_{B}:\mathop{\sum }\limits_{i}{B}_{ij}^{2}-1=0\end{array}$$

The total loss function is the weighted sum of the following losses:

9 $${{{{{{{\mathcal{L}}}}}}}}={\alpha }_{1}({{{{{{{{\mathcal{L}}}}}}}}}_{{{{{{{{\rm{AE}}}}}}}}}+{{{{{{{{\mathcal{L}}}}}}}}}_{{{{{{{{\rm{pred}}}}}}}}})+{\alpha }_{2}{{{{{{{{\mathcal{L}}}}}}}}}_{{{{{{{{\rm{AR}}}}}}}}}+{\alpha }_{3}{{{{{{{{\mathcal{L}}}}}}}}}_{{{{{{{{\rm{C}}}}}}}}}+{\alpha }_{4}\parallel {{{{{{{\bf{W}}}}}}}}{\parallel }_{2}^{2},$$

where

10 $${{{{{{{{\mathcal{L}}}}}}}}}_{{{{{{{{\rm{AE}}}}}}}}}=\parallel {{{{{\bf{x}}}}}}-{\phi }^{-1}(\phi ({{{{{{{\bf{x}}}}}}}})){\parallel }^{2},$$

11 $${{{{{{{{\mathcal{L}}}}}}}}}_{{{{{{{{\rm{pred}}}}}}}}}=\parallel {{{{{{{{\bf{x}}}}}}}}}_{{{{{{{{\bf{n+1}}}}}}}}}-{\phi }^{-1}\left\{{{{{{{{\bf{B}}}}}}}}\xi \left[{{{{{{{\bf{A}}}}}}}}\phi ({{{{{{{\bf{x}}}}}}}})\right]\right\}{\parallel }^{2},$$

12 $${{{{{{{{\mathcal{L}}}}}}}}}_{{{{{{{{\rm{AR}}}}}}}}}=\parallel {{{{{{{\bf{A}}}}}}}}\phi ({{{{{{{{\bf{x}}}}}}}}}_{n+1})-\xi \left[{{{{{{{\bf{A}}}}}}}}\phi ({{{{{{{{\bf{x}}}}}}}}}_{n})\right]{\parallel }^{2},$$

13 $${{{{{{{{\mathcal{L}}}}}}}}}_{{{{{{{{\rm{C}}}}}}}}}={C}_{AB}=\parallel {{{{{{{\bf{A}}}}}}}}\cdot {{{{{{{\bf{B}}}}}}}}-{{{{{{{\bf{I}}}}}}}}{\parallel }_{2}^{2}.$$

Here, ${{{{{{{{\mathcal{L}}}}}}}}}_{{{{{{{{\rm{AE}}}}}}}}}$ is the AE reconstruction loss, ${{{{{{{{\mathcal{L}}}}}}}}}_{{{{{{{{\rm{pred}}}}}}}}}$ is the future state reconstruction loss, ${{{{{{{{\mathcal{L}}}}}}}}}_{{{{{{{{\rm{AR}}}}}}}}}$ the auto-regression loss, ${{{{{{{{\mathcal{L}}}}}}}}}_{{{{{{{{\rm{C}}}}}}}}}$ is the loss to force the constraints from Eq. (8), and the term $\parallel {{{{{{{\bf{W}}}}}}}}{\parallel }_{2}^{2}$ is *L*2 regularization of NN weights to avoid over-fitting issue.

The weights *α*_1_, *α*_3_, *α*_4_ were assigned to the values of 1, 100, and 0.01, respectively. The weight *α*_2_ was gradually increased from 0 to 1 during training. The model was trained for 600 epochs with a learning rate of 0.001 and Adam optimizer^68^. The last 200 epochs were trained with a learning rate of 0.0001. The AE/AR NN architecture was implemented with Python and TensorFlow framework^69^.

The non-linear dynamics of the order parameter are crucial for explaining mortality. At the same time, the effects of the nonlinearity can be neglected almost always in the course of the life of an animal if the dimensionless parameter expressing the animal lifespan $\bar{t}$ in units of the mortality rate doubling time is large, $\alpha \bar{t}\, \gg \,1$^18^. Given the observed dFI doubling rates in mice, $\alpha \bar{t} \sim 3$ and hence the linear AR model is only a reasonable approximation. One should obtain better dFI variants in the future by increasing the rank in AR models, possibly including the effects of mode coupling with dFI.

### Model evaluation

The model was validated in test datasets (see Supplementary Data 3), which were completely excluded from the training of the AE-AR model. The test datasets were obtained from independent experiments by collecting CBC samples from cohorts of NIH Swiss mice of different age and sex (dataset MA0071), cohort NIH Swiss male mice observed for 15 months (dataset MA0072), and cohorts of naive male and female NIH Swiss mice that were humanely euthanized after reaching approved experimental endpoints (dataset MA0073).

We estimated the reconstruction error of the AE by calculation of the root mean squared error (RMSE) and the coefficient of determination *R*^2^ for each CBC feature in training and test sets (Supplementary Data 4 and Supplementary Data 5). The average RMSE in the test set was 229.6 with *R*^2^ = 0.55; in the training set, RMSE was 106.4 and *R*^2^ = 0.77. The best reconstruction was achieved for hematocrit (*R*^2^ = 0.95), red blood cells (*R*^2^ = 0.92) and lymphocytes (*R*^2^ = 0.87); the worst results were for mean corpuscular hemoglobin concentration (*R*^2^ = − 0.82) and platelets (*R*^2^ = − 0.14) in the test set. We note that according to definition, −1 < *R*^2^ < 1 (see, e.g.,^70^), the quantity may be negative in either the train or validation sets, which indicates cases of particularly bad fit.

### Determination of the effects of a drug on dFI

We performed the investigation of the effects of rapamycin on dFI trajectories in individual animals. Technically, we compared the increments of dFI levels along the individual life histories in time intervals depending on the amount of treatment between the subsequent time points. Such analysis explicitly relies on the equation of motion (7) for the order parameter, which is approximated by dFI. The drug’s effect manifests itself as the “force” term reducing the dFI increments between the measurements when the drug is given and having no effect (no force) whenever the drug is not administered, both in the treatment and control groups.

The determination of a drug’s effect on the aging process is therefore equivalent to determining the “force” term in the autoregression problem in Eq. (4). Since the natural variation of dFI levels between animals is often high, longitudinal studies should have more statistical power than standard group comparisons. The dFI was trained with the AR model (7). Accordingly, it is well suited to maximize the signal-to-noise ratio in a longitudinal analysis of an anti-aging intervention’s effects. If required, the autoregression model can accommodate any number of confounding factors, such as the experimental batch or sex of the animals. Technically, one can achieve the goal by adding the respective covariates to Eq’s right-hand side (4).

### Late-life mortality and survival analysis

The data for mice mortality were taken from ref. 25. Only control groups were selected for the analysis. Mice removed from the study were also removed from the current survival analysis. The mice were pooled together from all three study centers and cohorts and separated into two groups according to sex. Overall, there were 3249 male mice, and 2978 female mice.

The mortality analysis is done with the help of the Nelson-Aalen fitter from the *lifelines* python package^65^. For the Gompertz fit of the survivals and other survival analysis, we used the custom code published on GitHub.

### Reporting summary

Further information on research design is available in the Nature Research Reporting Summary linked to this article.

## Supplementary information

Supplementary Information

Description of Additional Supplementary Information

Supplementary Data 1

Supplementary Data 2

Supplementary Data 3

Supplementary Data 4

Supplementary Data 5

Supplementary Data 6

Reporting Summary

## Data Availability

The data supporting the findings of this study are available at the MPD (RRID:SCR_003212). Raw data files and scripts to reproduce all findings are available on GitHub https://github.com/gero-science/mice_dfi^71^. Additional data are available from the corresponding authors on reasonable request. Source data are provided with this paper.

## Source data

Source Data

## Code availability

The code will be available on GitHub https://github.com/gero-science/mice_dfi^71^

## References

[1] Horvath S (2013). DNA methylation age of human tissues and cell types. Genome Biol..

[2] Levine ME (2018). An epigenetic biomarker of aging for lifespan and healthspan. Aging.

[3] Antoch MP (2017). Physiological frailty index (pfi): quantitative in-life estimate of individual biological age in mice. Aging.

[4] Putin E (2016). Deep biomarkers of human aging: application of deep neural networks to biomarker development. Aging.

[5] Pyrkov, T. V. et al. Longitudinal analysis of blood markers reveals progressive loss of resilience and predicts ultimate limit of human lifespan. *Nat. Commun.* **12**, 2765 (2019). 10.1038/s41467-021-23014-1 PMC8149842 34035236

[6] Schultz MB (2020). Age and life expectancy clocks based on machine learning analysis of mouse frailty. Nat. Commun..

[7] Cohen AA (2013). A novel statistical approach shows evidence for multi-system physiological dysregulation during aging. Mechanisms Ageing Dev..

[8] Mitnitski, A. B., Mogilner, A. J. & Rockwood, K. Accumulation of deficits as a proxy measure of aging. *ScientificWorldJournal* **1**, 323–336 (2001). 10.1100/tsw.2001.58 PMC6084020 12806071

[9] Mitnitski A (2015). Age-related frailty and its association with biological markers of ageing. BMC Med..

[10] Blodgett JM, Theou O, Mitnitski A, Howlett SE, Rockwood K (2019). Associations between a laboratory frailty index and adverse health outcomes across age and sex. Aging Med..

[11] Justice J (2019). Senolytics in idiopathic pulmonary fibrosis: results from a first-in-human, open-label, pilot study. ebiomedicine.

[12] Fahy GM (2019). Reversal of epigenetic aging and immunosenescent trends in humans. Aging Cell.

[13] Hermann Haken. *Phys. Astron. online Libr*. 10.1007/978-3-662-10184-1 (Springer, Berlin, Heidelberg, 2004).

[14] Scheffer M (2009). Early-warning signals for critical transitions. Nature.

[15] Scheffer M (2018). Quantifying resilience of humans and other animals. Proc. Natl Acad. Sci. USA.

[16] Krotov D, Dubuis JO, Gregor T, Bialek W (2014). Morphogenesis at criticality. Proc. Natl Acad. Sci..

[17] Kogan V, Molodtsov I, Menshikov LI, Reis RJS, Fedichev P (2015). Stability analysis of a model gene network links aging, stress resistance, and negligible senescence. Sci. Rep..

[18] Podolskiy, D. Critical dynamics of gene networks is a mechanism behind ageing and Gompertz law. *arXiv* https://arxiv.org/abs/1502.04307 (2015).

[19] Bogue MA (2016). Accessing data resources in the mouse phenome database for genetic analysis of murine life span and health span. J. Gerontology. Ser. A.

[20] Fiedler, B. E. *Handbook of Dynamical Systems*, Vol. 2 (Gulf Professional Publishing, 2002).

[21] Seydel, R. *Practical Bifurcation and Stability Analysis*, Vol. 1, p. 477 (Springer Science & Business Media, 2009).

[22] Hughes BG, Hekimi S (2016). Different mechanisms of longevity in long-lived mouse and Caenorhabditis elegans mutants revealed by statistical analysis of mortality rates. Genetics.

[23] Yuan R (2012). Genetic coregulation of age of female sexual maturation and lifespan through circulating igf1 among inbred mouse strains. Proc. Natl Acad. Sci..

[24] Yuan R (2009). Aging in inbred strains of mice: study design and interim report on median lifespans and circulating igf1 levels. Aging Cell.

[25] Harrison DE (2009). Rapamycin fed late in life extends lifespan in genetically heterogeneous mice. Nature.

[26] O’Connell KE (2015). Practical murine hematopathology: a comparative review and implications for research. Comp. Med..

[27] Patel KV (2010). Red Cell distribution width and mortality in older adults: a meta-analysis. J. Gerontol..

[28] Baggiolini, M. Chemokines and leukocyte traffic. 10.1038/33340 (1998). 10.1038/33340 9560152

[29] Harris TB (1999). Associations of elevated interleukin-6 and C-reactive protein levels with mortality in the elderly. Am. J. Med..

[30] Mahmoudi S, Xu L, Brunet A (2019). Turning back time with emerging rejuvenation strategies. Nat. Cell Biol..

[31] Kuilman T, Michaloglou C, Mooi WJ, Peeper DS (2010). The essence of senescence. Genes Dev..

[32] van Deursen JM (2014). The role of senescent cells in ageing. Nature.

[33] Hall BM (2016). Aging of mice is associated with p16(Ink4a)- and *β*-galactosidasepositive macrophage accumulation that can be induced in young mice by senescent cells. Aging.

[34] Kim WY, Sharpless NE (2006). The regulation of INK4/ARF in cancer and aging. Cell.

[35] Burd CE (2013). Monitoring tumorigenesis and senescence in vivo with a p16 INK4a-luciferase model. Cell.

[36] Wilkinson JE (2012). Rapamycin slows aging in mice. Aging Cell.

[37] Bitto A (2016). Transient rapamycin treatment can increase lifespan and healthspan in middle-aged mice. elife.

[38] Miller RA (2014). Rapamycin-mediated lifespan increase in mice is dose and sex dependent and metabolically distinct from dietary restriction. Aging Cell.

[39] Balleza E (2008). Critical dynamics in genetic regulatory networks: examples from four kingdoms. PLoS One.

[40] Nakamura E, Miyao K, Ozeki T (1988). Assessment of biological age by principal component analysis. Mech. Ageing Dev..

[41] Park J, Cho B, Kwon H, Lee C (2009). Developing a biological age assessment equation using principal component analysis and clinical biomarkers of aging in korean men. Arch. Gerontol. geriatrics.

[42] Tarkhov AE (2019). A universal transcriptomic signature of age reveals the temporal scaling of caenorhabditis elegans aging trajectories. Sci. Rep..

[43] Johnstone IM, Lu AY (2009). On consistency and sparsity for principal components analysis in high dimensions. J. Am. Stat. Assoc..

[44] Mardt A, Pasquali L, Wu H, Noé F (2018). VAMPnets for deep learning of molecular kinetics. Nat. Commun..

[45] Lusch B, Kutz JN, Brunton SL (2018). Deep learning for universal linear embeddings of nonlinear dynamics. Nat. Commun..

[46] Wu T, Tegmark M (2019). Toward an artificial intelligence physicist for unsupervised learning. Phys. Rev. E.

[47] Liu, Z. & Tegmark, M. Ai poincar\’e: machine learning conservation laws from trajectories. *Phys. Rev. Lett.* **126**, 180604 (2020). 10.1103/PhysRevLett.126.180604 34018805

[48] Hofmann, B. Young blood rejuvenates old bodies: a call for reflection when moving from mice to men. *Karger* **1**, 45 (2018). 10.1159/000481828 PMC5836258 29593463

[49] Beerman I (2013). Proliferation-dependent alterations of the DNA methylation landscape underlie hematopoietic stem cell aging. Cell Stem Cell.

[50] Franceschi C (2006). Inflamm-aging: an evolutionary perspective on immunosenescence. Ann. N. Y. Acad. Sci..

[51] Pawelec G (2017). Immunosenescence and cancer. Biogerontology.

[52] Crooke, S. N., Ovsyannikova, I. G., Poland, G. A. & Kennedy, R. B. Immunosenescence and human vaccine immune responses. *Immun. Ageing* **16**, 25 (2019). 10.1186/s12979-019-0164-9 PMC6743147 31528180

[53] Pang WW (2011). Human bone marrow hematopoietic stem cells are increased in frequency and myeloid-biased with age. Proc. Natl Acad. Sci. USA.

[54] López-Otín C, Blasco MA, Partridge L, Serrano M, Kroemer G (2013). The hallmarks of aging. Cell.

[55] Partridge, L., Fuentealba, M. & Kennedy, B. K. The quest to slow ageing through drug discovery. *Nat. Rev. Drug Discov.* **19**, 513–532 (2020). 10.1038/s41573-020-0067-7 32467649

[56] Wang Y (2019). Prediction of chemotherapy adverse reactions and mortality in older patients with primary lung cancer through frailty index based on routine laboratory data. Clin. Interv. Aging.

[57] Kane AE, Keller KM, Heinze-Milne S, Grandy SA, Howlett SE (2019). A murine frailty index based on clinical and laboratory measurements: links between frailty and pro-inflammatory cytokines differ in a sex-specific manner. J. Gerontol. Ser. A.

[58] Sacher G, Trucco E (1962). The stochastic theory of mortality. Ann. N. Y. Acad. Sci..

[59] Yashin AI, Manton KG, Vaupel JW (1985). Mortality and aging in a heterogeneous population: a stochastic process model with observed and unobserved variables. Theor. Popul. Biol..

[60] Vaupel JW (1998). Biodemographic trajectories of longevity. Science.

[61] Pyrkov TV, Sokolov IS, Fedichev PO (2021). Deep longitudinal phenotyping of wearable sensor data reveals independent markers of longevity, stress, and resilience. Aging.

[62] Tarkhov, A. E., Denisov, K. A., & Fedichev, P. O. Aging clocks, entropy, and the limits of age-reversal. *bioRxiv* (McGraw-Hill, 2022).

[63] Pedregosa F (2011). Scikit-learn: machine learning in {P}ython. J. Mach. Learn. Res..

[64] Peterset, L. L. al. Large-scale, high-throughput screening for coagulation and hematologic phenotypes in mice. *Physiol. Genomics*.**11**, 185–193 (2002). 10.1152/physiolgenomics.00077.2002 12419856

[65] Davidson-Pilon, C. Lifelines. https://github.com/camdavidsonpilon/lifelines (2016)

[66] Hinton GE, Salakhutdinov RR (2006). Reducing the dimensionality of data with neural networks. Science.

[67] He, K., Zhang, X., Ren, S. & Sun, J. Deep residual learning for image recognition. in *2016 IEEE Conference on Computer Vision and Pattern Recognition (CVPR)*, 2016, pp. 770–778 (IEEE Computer Society, 2016).

[68] Kingma, D. P. & Ba, J. L. Adam: a method for stochastic optimization. https://arxiv.org/abs/1412.6980 (2014).

[69] Abadi, M. TensorFlow: large-scale machine learning on heterogeneous systems. https://www.tensorflow.org/ (2015).

[70] Steel, R. G. D. & Torrie, J. H. Principles and procedures of statistics: with special reference to the biological sciences. (McGraw Hill, 1960).

[71] Avchaciov, K. AI for unsupervised learning of aging principles from longitudinal data. 10.5281/zenodo.6991110 (2022). 10.1038/s41467-022-34051-9 PMC9626636 36319638

