# Supplementary material for: Unsupervised learning of aging principles from longitudinal data
Source: Nat Commun. 2022 Nov 1;13:6529. doi: 10.1038/s41467-022-34051-9 (PMC9626636; doi:10.1038/s41467-022-34051-9)
Supplement: Supplementary file 1 — Supplementary Information [file 41467_2022_34051_MOESM1_ESM.pdf]

Unsupervised learning of aging principles from longitudinal data

Konstantin Avchaciov,[1] Marina P. Antoch,[2] Ekaterina L. Andrianova,[3] Andrei E. Tarkhov,[1] Leonid I. Menshikov,[1] Olga Burmistrova,[1] Andrei V. Gudkov,[3,4] and Peter O. Fedichev[1,*]

[1]*Gero PTE. LTD., 409051, Singapore*
[2]*Department of Pharmacology and Therapeutics, Roswell Park Comprehensive Cancer Center, Buffalo, NY*
[3]*Genome Protection, Inc., Buffalo, NY*
[4]*Department of Cell Stress Biology, Roswell Park Comprehensive Cancer Center, Buffalo, NY*

[*] Correspondence and requests for materials should be addressed to P.O.F. email peter.fedichev@gero.ai

SUPPLEMENTARY FIGURES

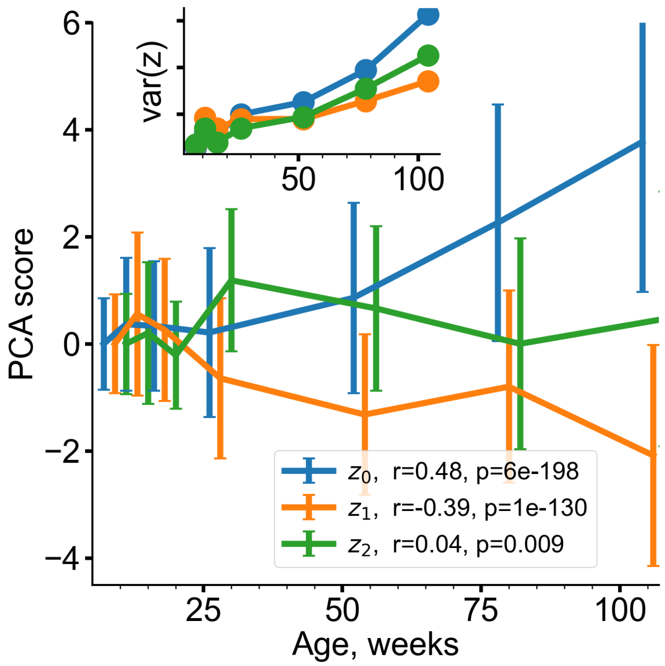

Supplementary Fig. 1: **Principal Component (PC) Analysis of complete blood count measurements from the Mouse Phenome Database data (including young animals).** The graphs represent the average PC scores in subsequent age groups (the error bars are standard deviations). The inset shows that the variance for all PC scores increases with age. Two-sided p values were calculated for the Pearson correlation coefficient between PC scores and age in the sample size of $n = 3507$ animals.

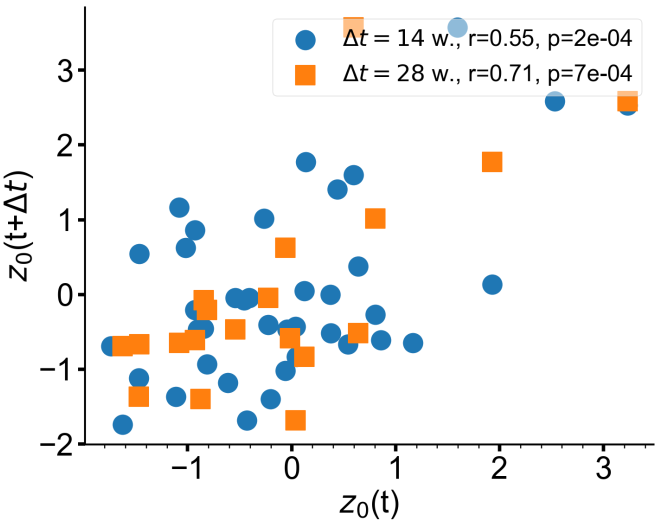

Supplementary Fig. 2: **Auto-correlation property of the first primary component (PC, z_0).** Correlation between age-adjusted the first PC score (z_0) across sampling intervals Δt of 14 (blue circles, animals $n = 40$) and 28 (orange squares, animals $n = 19$) weeks in the validation MA0072 dataset. Two-sided p values were calculated for the Pearson correlation coefficient r.

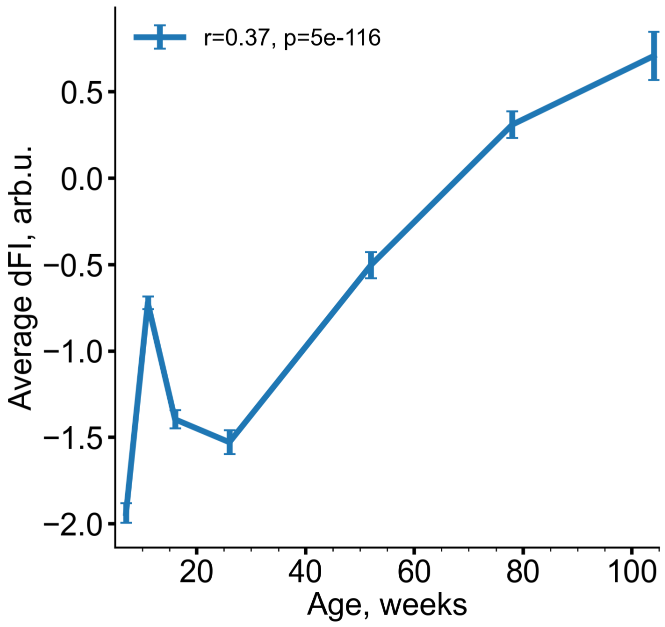

Supplementary Fig. 3: **The dynamic frailty indicator (dFI) as a function of age in the training dataset.** All data are presented as Mean±SEM. Two-sided p values were calculated for the Pearson correlation coefficient r in the sample size of $n = 3507$ animals.

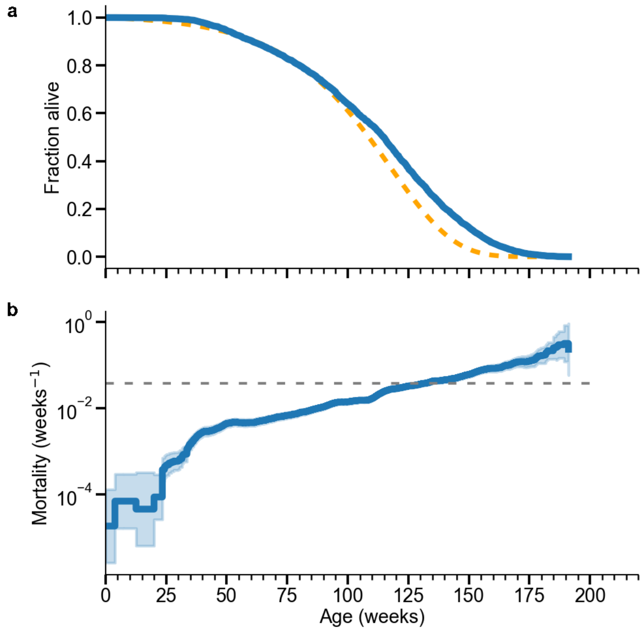

Supplementary Fig. 4: **Late-life mortality deceleration in male mice cohort.** (**a**) The Kaplan-Meier survival curve (solid blue line) in male mice cohort from [1]. The orange dashed line represents the best Gompertz fits. (**b**) The Nelson-Aalen estimator of total mortality (solid blue line) and the 95% confidence intervals are filled in blue. The grey dashed line corresponds to the mortality level according to the theoretical prediction $M(t \gg \bar{t}) = \alpha$.

.

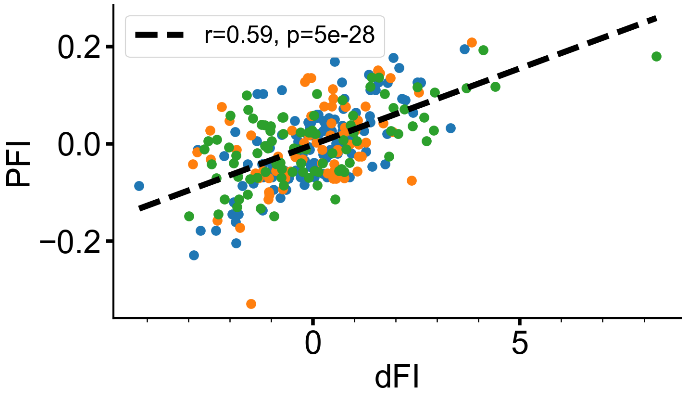

Supplementary Fig. 5: **Correlation between the dFI and the physiological frailty index (PFI) after adjustment for sex and age**. Colors represent animals from the test dataset, where blue, orange and green circles are females in MA0071, males in MA0071 and males in MA0072, respectively. Two-sided p values were calculated for the Pearson correlation coefficient r.

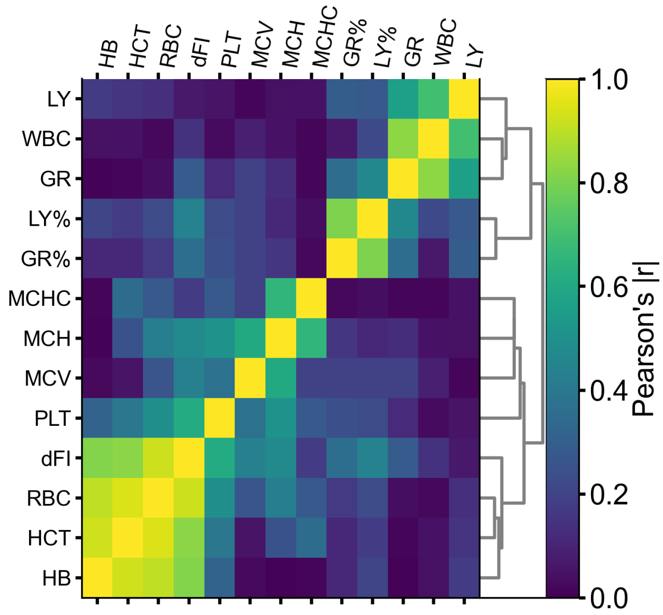

Supplementary Fig. 6: **Hierarchical clustering of CBC features and dFI score in the test dataset.** The colors represent the Pearson's correlation coefficient (absolute value) as indicated by the scale on the right.

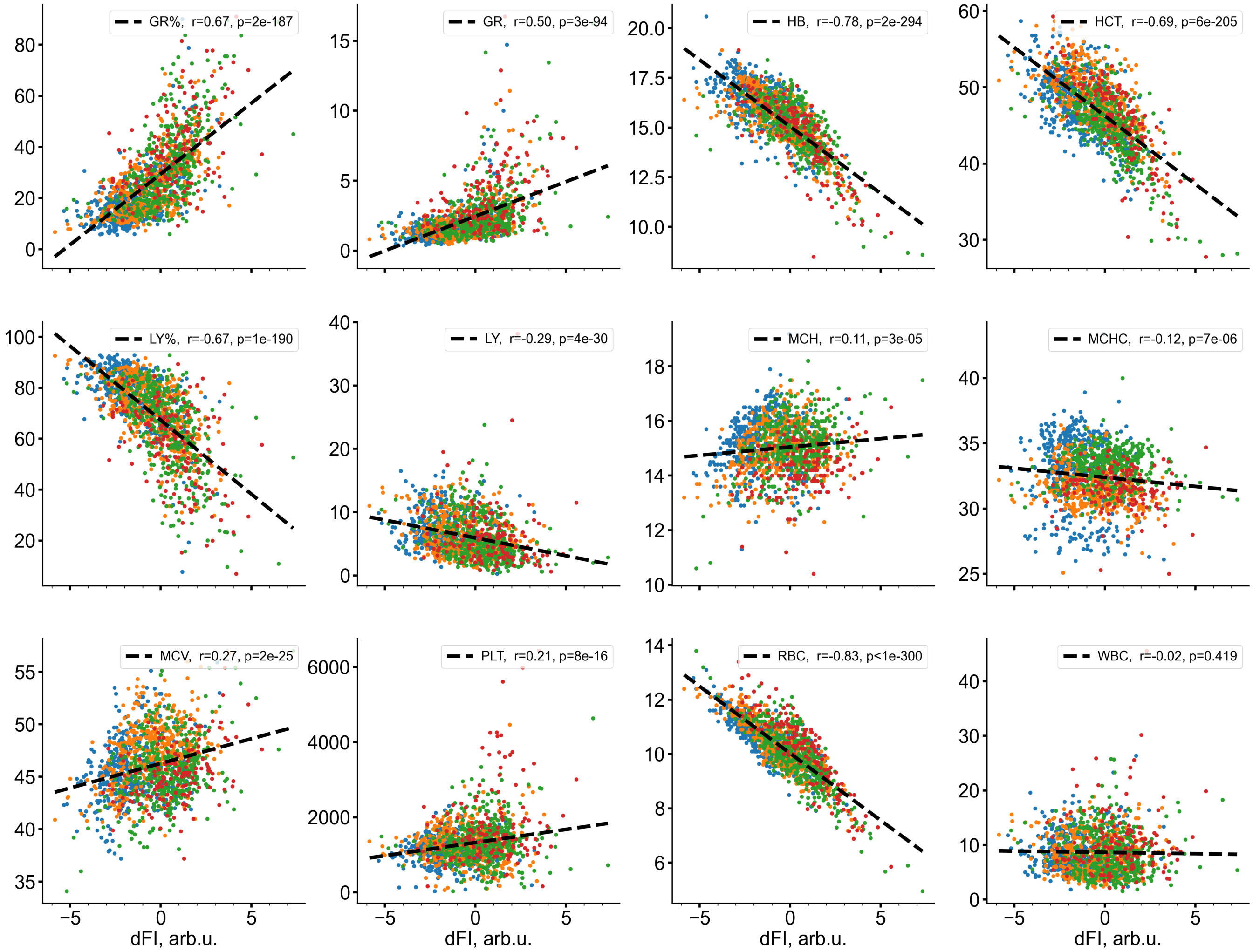

Supplementary Fig. 7: **Correlations between dFI and CBC parameters in the *Peters4* dataset.** The horizontal and vertical axis denotes dFI and CBC feature. The sub-figure's legend includes the feature name, the Pearson coefficient of correlation, and p value (two-sided, sample size $n = 1485$ animals). Orange, green, and blue colors represent the measurements from animals in the age groups 26, 52, 78 and 104 weeks old, respectively.

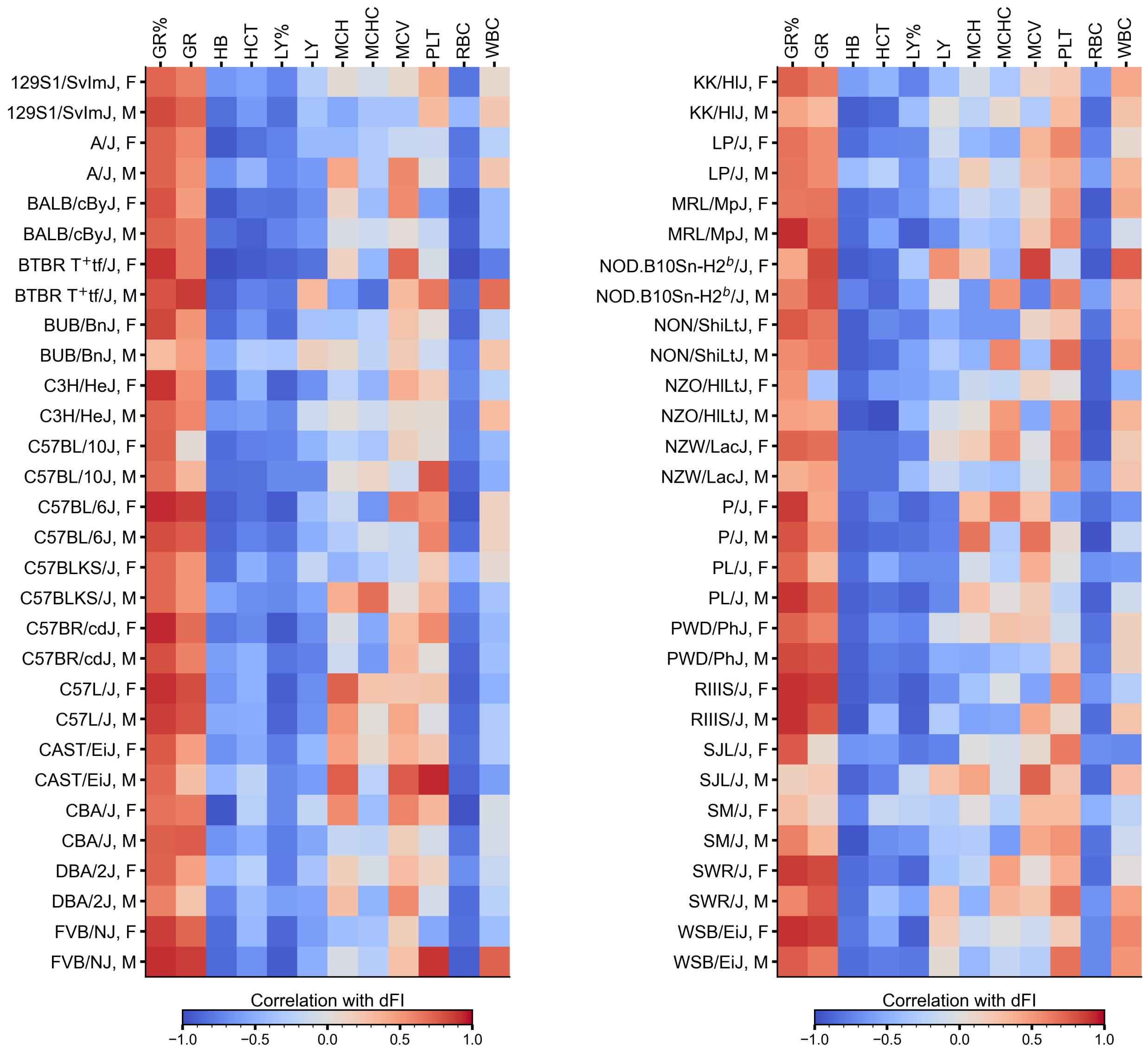

Supplementary Fig. 8: **The correlation coefficient between dFI and individual CBC features is different (sometimes even of opposite sign) in various mouse strains.** Pearson correlations coefficient between dFI and CBC parameters in the *Peters4* dataset shown for cohorts of mice of same strain and sex.

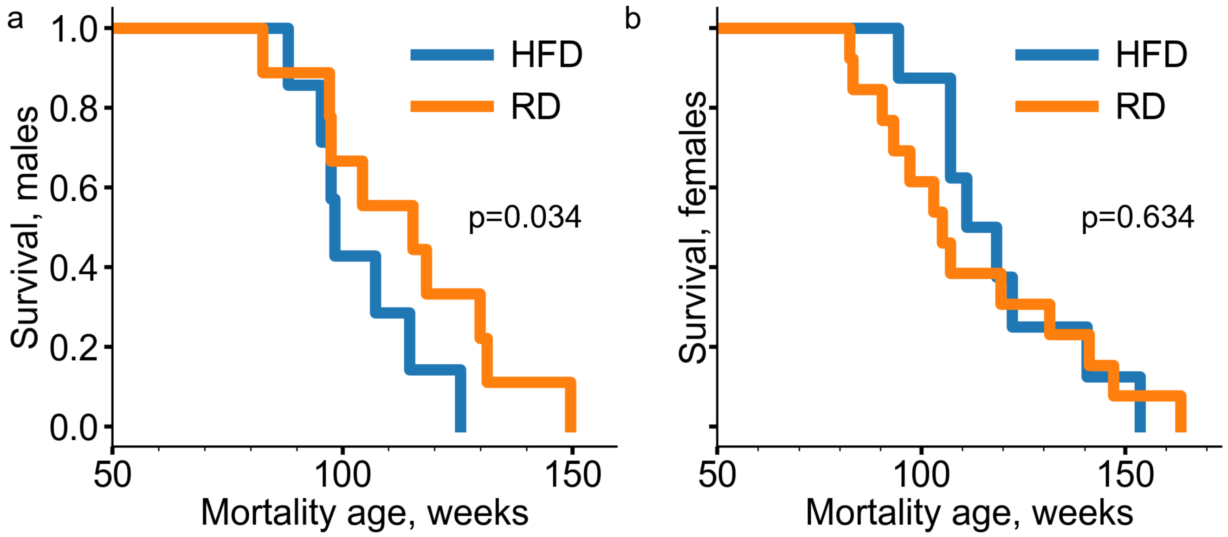

Supplementary Fig. 9: **Effects of life-long treatment on the lifespan and survival.** Kaplan-Meier survival curves showing that long-term (26 weeks) high fat diet (HFD) feeding significantly reduces the lifespan of male (**a**) (p value $= 0.034$, log-rank test), but not female (**b**) ($p = 0.6$, log-rank test) mice in comparison with regular diet (RD) feeding. Data is taken from Antoch et.al, 2017 [2] and re-published with permission from authors.

[1] D. E. Harrison, R. Strong, Z. D. Sharp, J. F. Nelson, C. M. Astle, K. Flurkey, N. L. Nadon, J. E. Wilkinson, K. Frenkel, C. S. Carter, M. Pahor, M. A. Javors, E. Fernandez, R. A. Miller, and Others, Rapamycin fed late in life extends lifespan in genetically heterogeneous mice, Nature **460**, 392 (2009).
[2] M. P. Antoch, M. Wrobel, K. K. Kuropatwinski, I. Gitlin, K. I. Leonova, I. Toshkov, A. S. Gleiberman, A. D. Hutson, O. B. Chernova, and A. V. Gudkov, Physiological frailty index (pfi): quantitative in-life estimate of individual biological age in mice, Aging (Albany NY) **9**, 615 (2017).
