# Supplementary material for: Unsupervised learning of aging principles from longitudinal data
Source: Nat Commun. 2022 Nov 1;13:6529. doi: 10.1038/s41467-022-34051-9 (PMC9626636; doi:10.1038/s41467-022-34051-9)
Supplement: Supplementary file 2 — Description of Additional Supplementary Information [file 41467_2022_34051_MOESM2_ESM.docx]

# Description of Additional Supplementary Files

File Name: Supplementary Data 1

Description: **The total number of animals in the Mouse Phenome Database (MPD) datasets used for Principal Component Analysis and neural network training**.

File Name: Supplementary Data 2

Description: **List of abbreviations and acronyms used in this article.**

File Name: Supplementary Data 3

Description: **Total number of animals in the test dataset.**

File Name: Supplementary Data 4

Description: **Performance evaluation of the autoencoder in the training dataset**. Reconstruction error (root-mean-square error, RMSE) and coefficient of determination, R^2, were calculated for each complete blood count component in the training set.

File Name: Supplementary Data 5

Description: **Performance evaluation of the autoencoder in the test dataset**. Reconstruction error (root-mean-square error, RMSE) and coefficient of determination, R^2, were calculated for each complete blood count component in the test set.

File Name: Supplementary Data 6

Description: **The dynamic frailty indicator (dFI) predicts the remaining lifespan**. Univariate Cox proportional hazards model hazard ratio (HR) for animals of the same age and sex. Values in parentheses are HR 95% confidence interval and *p*-value. The analysis is shown for two cohorts: Cohort 1 includes all animals with mortality data, and Cohort 2 includes the subset of animals from Cohort 1 for which IGF1 measurements were available. HR_CBC_ denotes to log-hazard ratio trained from complete blood counts in the same dataset as dFI. Statistically significant hazard ratios (*p*<0.05) are highlighted in bold.
