# Supplementary material for: Unsupervised learning of aging principles from longitudinal data
Source: Nat Commun. 2022 Nov 1;13:6529. doi: 10.1038/s41467-022-34051-9 (PMC9626636; doi:10.1038/s41467-022-34051-9)
Supplement: Supplementary file 9 — Reporting Summary [file 41467_2022_34051_MOESM9_ESM.pdf]

nature portfolio

Corresponding author(s): Konstantin Avchaciov, Peter Fedichev

Last updated by author(s): Aug 15, 2022

Reporting Summary

Nature Portfolio wishes to improve the reproducibility of the work that we publish. This form provides structure for consistency and transparency in reporting. For further information on Nature Portfolio policies, see our Editorial Policies and the Editorial Policy Checklist.

Statistics

For all statistical analyses, confirm that the following items are present in the figure legend, table legend, main text, or Methods section.

n/a	Confirmed	
☐	☒	The exact sample size (*n*) for each experimental group/condition, given as a discrete number and unit of measurement
☒	☐	A statement on whether measurements were taken from distinct samples or whether the same sample was measured repeatedly
☐	☒	The statistical test(s) used AND whether they are one- or two-sided<br>*Only common tests should be described solely by name; describe more complex techniques in the Methods section.*
☐	☒	A description of all covariates tested
☒	☐	A description of any assumptions or corrections, such as tests of normality and adjustment for multiple comparisons
☐	☒	A full description of the statistical parameters including central tendency (e.g. means) or other basic estimates (e.g. regression coefficient) AND variation (e.g. standard deviation) or associated estimates of uncertainty (e.g. confidence intervals)
☐	☒	For null hypothesis testing, the test statistic (e.g. *F*, *t*, *r*) with confidence intervals, effect sizes, degrees of freedom and *P* value noted<br>*Give P values as exact values whenever suitable.*
☒	☐	For Bayesian analysis, information on the choice of priors and Markov chain Monte Carlo settings
☒	☐	For hierarchical and complex designs, identification of the appropriate level for tests and full reporting of outcomes
☐	☒	Estimates of effect sizes (e.g. Cohen's *d*, Pearson's *r*), indicating how they were calculated

Our web collection on statistics for biologists contains articles on many of the points above.

Software and code

Policy information about availability of computer code

Data collection	No software was used
Data analysis	python==3.8, tensorflow==2.6.0, scikit-learn==0.23.2,scipy==1.8.1, lifelines==0.26

For manuscripts utilizing custom algorithms or software that are central to the research but not yet described in published literature, software must be made available to editors and reviewers. We strongly encourage code deposition in a community repository (e.g. GitHub). See the Nature Portfolio guidelines for submitting code & software for further information.

Data

Policy information about availability of data

All manuscripts must include a data availability statement. This statement should provide the following information, where applicable:

- Accession codes, unique identifiers, or web links for publicly available datasets
- A description of any restrictions on data availability
- For clinical datasets or third party data, please ensure that the statement adheres to our policy

The data supporting the findings of this study are available at the Mouse Phenome Database (MPD, RRID:SCR_003212[https://phenome.jax.org]). Raw data files and scripts to reproduce all findings are available on GitHub https://github.com/gero-science/mice_dfi. Additional data are available from the corresponding authors on reasonable request. Source data are provided with this paper.

Human research participants

Policy information about studies involving human research participants and Sex and Gender in Research.

Reporting on sex and gender	not applicable
Population characteristics	not applicable
Recruitment	not applicable
Ethics oversight	not applicable

Note that full information on the approval of the study protocol must also be provided in the manuscript.

Field-specific reporting

Please select the one below that is the best fit for your research. If you are not sure, read the appropriate sections before making your selection.

☒ Life sciences ☐ Behavioural & social sciences ☐ Ecological, evolutionary & environmental sciences

For a reference copy of the document with all sections, see nature.com/documents/nr-reporting-summary-flat.pdf

Life sciences study design

All studies must disclose on these points even when the disclosure is negative.

Sample size	Resource equation method was applied. Size of 12 mice per group is sufficient to satisfy 10 < E < 20 criteria.
Data exclusions	No data were excluded.
Replication	Findings were not replicated due to high cost of the experiment. Also, the life-extending effect of rapamycin were discussed previously in literature (see discussion in the manuscript).
Randomization	Mice was divided into treatment and control groups using a stratified randomization technique to produce the indistinguishable distributions of dFI values prior to the experiment
Blinding	Blinding was not relevant to this study since the effect of therapies (high fat diet, rapamycin) was reported previously in the literature. This study has no conclusions regarding effects of therapies on animal lifespan.

Reporting for specific materials, systems and methods

We require information from authors about some types of materials, experimental systems and methods used in many studies. Here, indicate whether each material, system or method listed is relevant to your study. If you are not sure if a list item applies to your research, read the appropriate section before selecting a response.

Materials & experimental systems

n/a	Involved in the study	
☒	☐	Antibodies
☒	☐	Eukaryotic cell lines
☒	☐	Palaeontology and archaeology
☐	☒	Animals and other organisms
☒	☐	Clinical data
☒	☐	Dual use research of concern

Methods

n/a	Involved in the study	
☒	☐	ChIP-seq
☒	☐	Flow cytometry
☒	☐	MRI-based neuroimaging

Animals and other research organisms

Policy information about studies involving animals; ARRIVE guidelines recommended for reporting animal research, and Sex and Gender in Research

Laboratory animals	Dataset MA0071: male and female NIH Swiss mice, males age 26, 64, 78, 92 and 136 weeks, females age 30, 56, 68, 82, 95, 108 and 136 weeks

	Dataset MA0072: male NIH Swiss mice, age 66 weeks<br>Dataset MA0073: male and female NIH Swiss mice, ages 75-165 weeks<br>Dataset p16-Luc: p16/INK4a-LUC female mice, ages 44-106 weeks<br>Dataset Rapamycin, C57BL/6 male mice, age 60 weeks
Wild animals	The study did not involve wild animals
Reporting on sex	Most of our experiments involved control and treatment groups of animals of both sexes. The only exception was the experiment characterizing the effects of rapamycin treatment which only involved male animals. According to the Intervention Testing Program (10.1038/nature08221), rapamycin extends lifespan in both sexes. Therefore, we selected the animals of one sex to reduce the specimen number, housing, and the running cost of the experiment.
Field-collected samples	The study did not involve samples collected from the field.
Ethics oversight	All animal experiments were approved by the Institutional Animal Care and Use Committee of Roswell Park Cancer Institute or by the Explora BioLabs, Inc Animal Use Committee.

Note that full information on the approval of the study protocol must also be provided in the manuscript.
